# InVision: An optimized tissue clearing approach for three-dimensional imaging and analysis of intact rodent eyes

**DOI:** 10.1016/j.isci.2021.102905

**Published:** 2021-07-26

**Authors:** Akshay Gurdita, Philip E.B. Nickerson, Neno T. Pokrajac, Arturo Ortín-Martínez, En Leh Samuel Tsai, Lacrimioara Comanita, Nicole E. Yan, Parnian Dolati, Nobuhiko Tachibana, Zhongda C. Liu, Joel D. Pearson, Danian Chen, Rod Bremner, Valerie A. Wallace

**Affiliations:** 1 Donald K. Johnson Eye Institute, Krembil Research Institute, University Health Network, 60 Leonard Avenue, Toronto, ON M5T 2S8, Canada; 2 Department of Laboratory Medicine and Pathobiology, University of Toronto, Toronto, ON M5S 1A8, Canada; 3 Lunenfeld-Tanenbaum Research Institute, Sinai Health System, Toronto, ON M5G 1X5, Canada; 4 Research Laboratory of Ophthalmology and Vision Sciences, State Key Laboratory of Biotherapy, Department of Ophthalmology, West China Hospital, Sichuan University, Chengdu, China; 5 Department of Ophthalmology and Vision Sciences, University of Toronto, Toronto, ON M5T 3A9, Canada

**Keywords:** Optical imaging, Biological sciences, Neuroscience, Biotechnology, Biological sciences research methodologies, Biology experimental methods

## Abstract

The mouse eye is used to model central nervous system development, pathology, angiogenesis, tumorigenesis, and regenerative therapies. To facilitate the analysis of these processes, we developed an optimized tissue clearing and depigmentation protocol, termed *InVision*, that permits whole-eye fluorescent marker tissue imaging. We validated this method for the analysis of normal and degenerative retinal architecture, transgenic fluorescent reporter expression, immunostaining and three-dimensional volumetric (3DV) analysis of retinoblastoma and angiogenesis. We also used this method to characterize material transfer (MT), a recently described phenomenon of horizontal protein exchange that occurs between transplanted and recipient photoreceptors. 3D spatial distribution analysis of MT in transplanted retinas suggests that MT of cytoplasmic GFP between photoreceptors is mediated by short-range, proximity-dependent cellular interactions. The *InVision* protocol will allow investigators working across multiple cell biological disciplines to generate novel insights into the local cellular networks involved in cell biological processes in the eye.

## Highlights

• *InVision* is an optimized tissue clearing protocol for the rodent eye
• *InVision* can be used to study a wide variety of physiological processes in the eye
• Material transfer between transplanted and host photoreceptors is spatially correlated

## Graphical abstract

**Figure:**
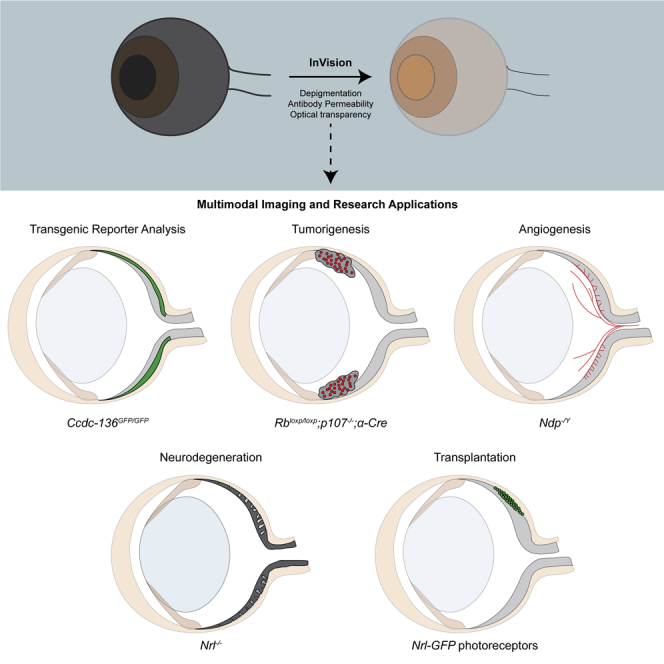

## Introduction

The mouse eye serves as a powerful tool to model the development, function, and diseases of the central nervous system (CNS) (London et al., 2013), in part because of the availability of a vast array of validated lineage-specific reporters and immunolabeling reagents. Advances in laser microscopy instrumentation, such as multiphoton (MP) and light sheet fluorescence microscopy (LSFM) improve accessibility to deeper, fluorescently labeled CNS structures, such as the brain, within higher volume samples without the need for classical histological sectioning. MP imaging utilizes higher penetrating, long-wavelength laser scanning to excite single optical planes in deeper structures, whereas LSFM uses an orthogonal plane of light (sheet) to excite a single optical section. Both methods provide the ability to view tissues with cellular resolution, although each modality harbors its own advantage. MP scanning generates high z-resolution, sub-cellular detail, but the speed of acquisition is limited by scanning galvanometer speeds. This limitation in acquisition speed makes large-volume imaging prohibitive and requires that the user assemble multiple volumetric z-stacks into 3D renderings using multidimensional stitching. In contrast, LSFM provides lower z-resolution images but has the advantage of fast acquisition of data within large volumes of tissue. The assembly of a methodological workflow wherein a single, intact ocular tissue sample can be serially imaged utilizing both MP and LSFM approaches would provide complementary datasets that capitalize on the advantages of each instrument.

Recently, the use of tissue clearing solvents and analytic protocols, such as clear, unobstructed brain/body imaging cocktails and computational analysis (CUBIC), have made MP and LSFM well-suited for deep imaging within large transparent tissues (Nojima et al., 2017; Susaki et al., 2014, 2015). Furthermore, the optical accessibility afforded by tissue clearing helps to minimize laser intensities required for signal detection, thus mitigating photo-bleaching. Collectively, these advances coalesce to provide investigators with the ability to generate 3D reconstructed images from optically sectioned whole tissue samples (Nojima et al., 2017; Susaki et al., 2014, 2015). This approach effectively eliminates sampling error due to artifacts derived from the analysis of serial tissue sections, and greatly enhances the throughput of biological imaging.

Multidisciplinary fields that use the eye as a model of CNS tissue, and those that study the eye directly in the context of vision science would benefit significantly from a protocol that could produce cleared, whole eyes for use with MP and LSFM platforms. The heterogeneity of the tissues that make up the eye limit the usefulness of original clearing protocols that were developed for non-pigmented and relatively homogeneous tissue types, such as the brain (Renier et al., 2014; Susaki et al., 2014; Susaki et al., 2015; Tomer et al., 2014). The cornea and sclera, for example, provide the eye with a dense, outer physical barrier that abates antibody and vital dye penetration. Moreover, highly pigmented structures, such as the iris and retinal pigmented epithelium, pose an optical barrier to laser excitation and detection of emitted photons. Several tissue clearing protocols, used with various imaging modalities to study diverse biological applications, have been recently developed for clearing eyes using modified versions of seeDB (Hohberger et al., 2017), ethanol-ECi (EyeCi) (Henning et al., 2019), iDISCO (EyeDISCO) (Vigouroux et al., 2020), PACT (Prahst et al., 2020) and CUBIC (Ye et al., 2020). In three of these studies, the problem posed by pigmented structures was addressed by incorporating similar melanin bleaching steps (Henning et al., 2019; Vigouroux et al., 2020; Ye et al., 2020). Moreover, to improve immunostaining efficiency, these protocols also require injecting antibodies into the eye (Henning et al., 2019; Ye et al., 2020), making an incision in the cornea (Vigouroux et al., 2020), or dissecting the eyecups (Prahst et al., 2020), thus mechanically disrupting the intact tissue. Finally, eyes may require embedding in agarose in some protocols (Henning et al., 2019; Prahst et al., 2020; Vigouroux et al., 2020) and this may limit downstream applications and imaging modalities. Consequently, variability in the depigmentation approaches, maintenance of tissue morphology, and the extent of antibody penetration across protocols result in varying degrees of efficiency. To resolve these challenges, we developed a tissue processing protocol for the mouse eye, termed *InVision*, that can be used to generate optically accessible, whole mouse eyes that are amenable to imaging of transgenic fluorescent reporters, as well as follow-up immunolabeling and vital dye counterstaining reactions. Our protocol includes modifications to the CUBIC clearing protocol (Nojima et al., 2017; Susaki et al., 2014, 2015), incorporation of depigmentation approaches, and protease treatment for enhanced antibody penetration. We demonstrate that *InVision* can be used to analyze a range of cell biological processes in the eye. Moreover, we demonstrate that intercellular protein transfer in a transplantation context is spatially dependent.

## Results

### Evaluation of tissue clearing, depigmentation and immunostaining protocols for whole rodent eyes

**Figure 1:**
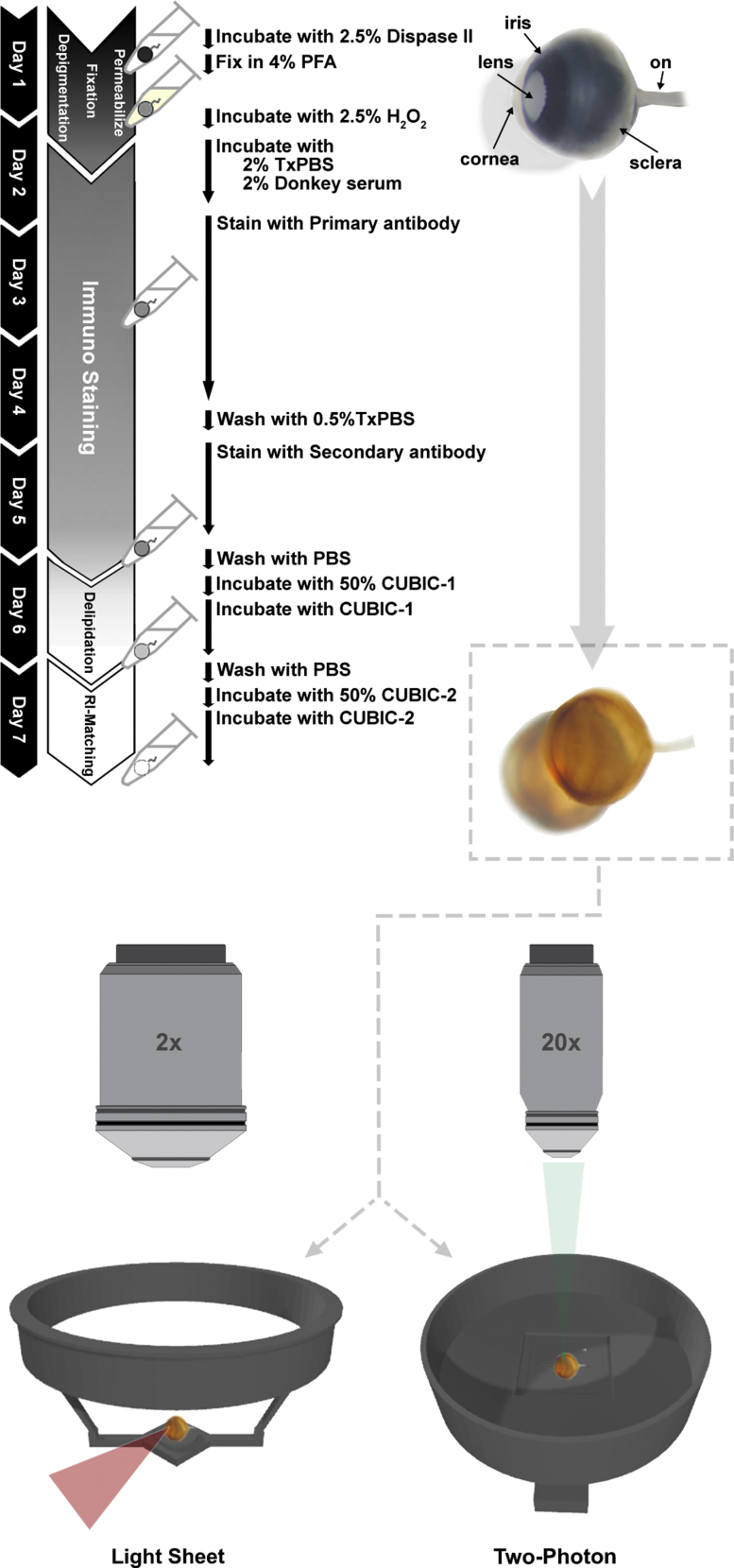
Overview of the InVision protocol InVision combines pre-processing steps with CUBIC clearing for clearing of the intact adult rodent eye. Enzymatic digestion of collagen rich structures using dispase allows for improved antibody permeability. Bleaching with hydrogen peroxide allows for imaging of pigmented eyes while maintaining the structural integrity of the tissue. CUBIC protocol incubation times have also been optimized for the size of the rodent eye; ON, optic nerve.

**Figure 2:**
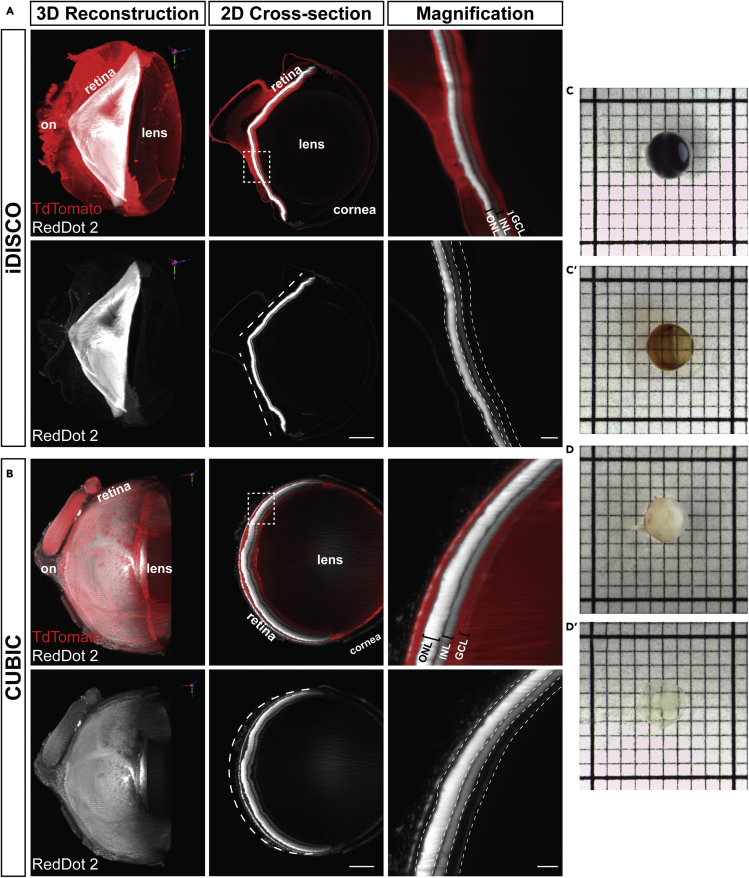
Modified CUBIC protocol allows for imaging of pigmented and albino eyes (A and B) Albino *ROSA*^*mT/mG*^ mouse eyes cleared using (A) iDISCO and (B) CUBIC clearing techniques. Organic solvent-based methods, such as iDISCO, result in significant shrinkage of the retina consequently causing the retina to collapse inwards and adversely affect the 3D tissue morphology. Dotted white lines in the 2D cross-section of the entire eye demonstrate the morphological change of the retina. The retina is shaped more like a ‘V’ after iDISCO compared to a more circular shape after CUBIC clearing. Magnified regions are from the white boxed area in the 2D cross-section. Scale bars, 2D cross section: 500 μm, magnification: 100 μm. (C and D) (C) Pigmented and (D) albino mouse eyes before (C and D) and after (C′ and D′) applying our clearing protocol. 1 mm grid. ONL, outer nuclear layer; INL, inner nuclear layer; GCL, ganglion cell layer; on, optic nerve. Dashed lines in magnification indicate layers of the retina.

There are a variety of established clearing methods for use with neural tissue, each differing in clearing time, impact on tissue morphology, ease of use, and compatibility with endogenous reporters and immunostaining (Richardson and Lichtman, 2015). We evaluated the effects of two established clearing methods, iDISCO (Renier et al., 2014) and CUBIC (Nojima et al., 2017; Susaki et al., 2014, 2015), on tissue integrity and staining compatibility in whole albino eyes from *ROSA*^*mT/mG*^ albino mice, a *Cre* reporter strain that expresses ubiquitous membrane associated dtTomato from the ROSA locus (Muzumdar et al., 2007). To adapt microscope stages to facilitate imaging of whole rodent eyes, we custom-designed and 3D-printed solvent-resistant ocular imaging inserts that permit the unobstructed multimodal imaging of immobilized rodent eyes (Figures 1, S1, S2A, and Data S1). Light sheet microscopic imaging of iDISCO- and CUBIC-cleared *ROSA*^*mT/mG*^ eyes revealed marked differences in tissue shrinkage and morphological disruption. Specifically, iDISCO induced the neural retina and sclera to distort relative to the cornea and lens (Figure 2A and Video S1). Embedding the eyes in agarose, as reported by other groups that have utilized dehydration-based approaches as the basis of clearing protocols for the eye (Henning et al., 2019; Vigouroux et al., 2020), may account for the differences in reported morphology. In contrast, the hyper-expansion clearing technique, CUBIC, resulted in a uniform expansion of all ocular tissues, resulting in an accurate approximation of eye structure (Figure 2B and Video S2). Furthermore, endogenous tdTomato fluorescence signal and tissue optical clarity permitted whole-organ light sheet imaging (Figure 2B), prompting us to employ the CUBIC clearing protocol as the basis of our *InVision* ocular clearing method.

Video S1. 3D reconstruction of an iDISCO-cleared mouse eye, related to Figure 2 3D reconstruction of an albino *ROSA*^*mT/mG*^ mouse eye cleared using iDISCO. TdTomato (red), RedDot2 (white).

Video S2. 3D reconstruction a CUBIC-cleared mouse eye, related to Figure 2 3D reconstruction of an albino *ROSA*^*mT/mG*^ mouse eye cleared using CUBIC. TdTomato (red), RedDot2 (white).

**Figure 3:**
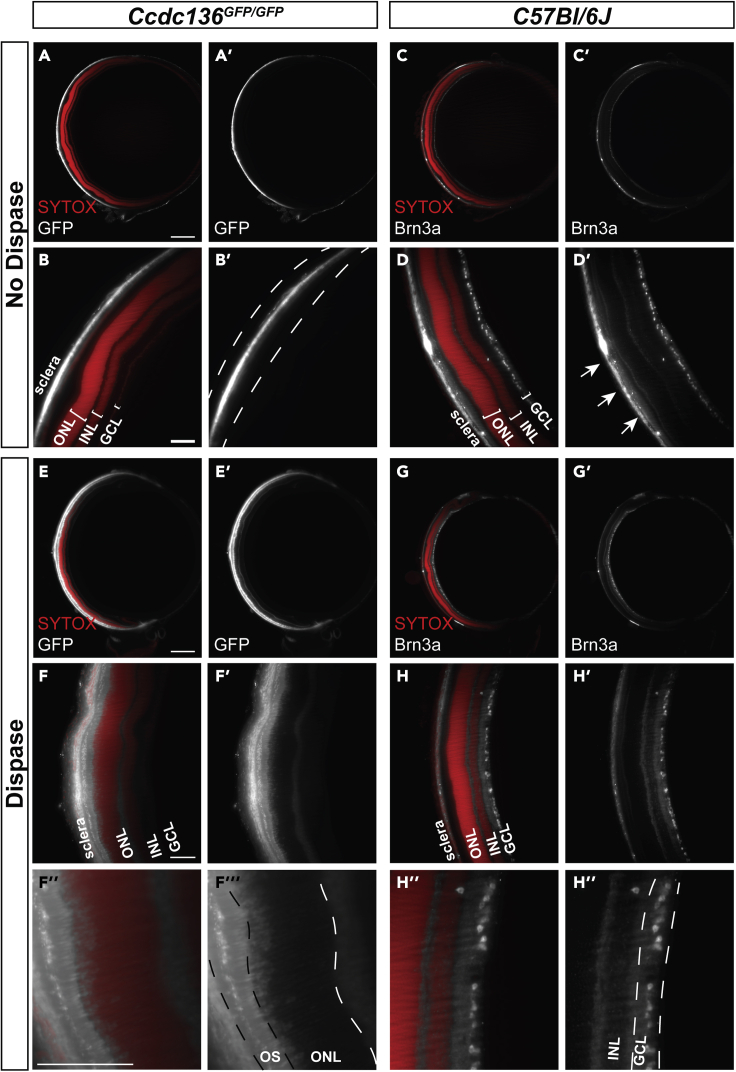
Dispase improves antibody penetration for immunolabeling of intact eyes Optical sections of pigmented (A, B, E, F) *Ccdc*-*GFP* or (C, D, G, H) C57BL/6J retinas immunostained with SYTOX Orange nuclear stain and anti-GFP or anti-Brn3a (A, B, C, D) without and (E, F, G, H) with dispase treatment. High background staining at the sclera is present between the dashed lines in B’. Arrows in D′ indicate high background staining at the sclera. ONL, outer nuclear layer; INL, inner nuclear layer; GCL, ganglion cell layer; OS, outer segment. Scale bars = 500 μm for A, C, E, G and 100 μm for B, D, F, and H.

Although effective in albino mouse eyes, we sought to determine whether CUBIC clearing is compatible with protocol modifications that remove melanin. We optimized concentration, time, and temperature variables for hydrogen-peroxide-based (H_2_O_2_) depigmentation of CUBIC-cleared, C57BL/6J mouse eyes, a pigmented strain (Figure 2C). Similar to CUBIC-cleared albino counterparts (Figures 2B and 2D), H_2_O_2_ depigmentation did not affect the ocular structure of C57BL/6J eyes (Figures 2C, 3A–3D, and S2B), indicating that this method is effective and compatible with CUBIC, but it quenched the endogenous GFP fluorescence in retinas of *Ccdc-136*^*GFP/GFP*^ (Ccdc-GFP) mice, a reporter strain with GFP expression in cone photoreceptors and bipolar cells (Smiley et al., 2016). Immunolabeling of cleared and H_2_O_2_ depigmented intact eyes with anti-GFP antibodies (Figure 3; Table 1) revealed no signal compared to immunolabeling of dissected retinas (Figure S2C). However, immunolabeling with anti-Brn3a, which labels ganglion cells in the inner retina, remained intact (Table 1 and Figures 3C and 3D). It is unclear why immunostaining in the intact eye is dependent on the primary antibody, although one explanation may be limited tissue penetration. That the intact eye hinders antibody penetration to the inner tissue is consistent with methods employed by other groups for immunostaining cleared eyes, including staining dissected eye cups (Prahst et al., 2020), antibody injection into the eye (Henning et al., 2019; Ye et al., 2020) or requiring a corneal incision (Vigouroux et al., 2020). We tested dispase treatment as an approach to digest collagens within the cornea, sclera, and Bruch’s membrane structures (Ihanamaki et al., 2004). The concentration, time, and temperature were again optimized to provide adequate antibody staining without significantly affecting the tissue structures in CUBIC-cleared, H_2_O_2_-depigmented C57BL/6J eyes (Figure 3). Dispase treatment of depigmented Ccdc-GFP eyes was sufficient to allow immunohistochemical recovery of GFP signal in cone photoreceptors in the outer nuclear layer (ONL), demonstrating a dispase-associated recovery of a peroxide-quenched endogenous reporter (Figures 3E and 3F). Moreover, dispase treatment reduced background at the sclera (Figure 3H’) compared to non-dispase treated eyes (Figure 3D’). Surprisingly, GFP expression in bipolar cells was not recovered. Thus, further antibody optimization may be required to recover lower levels of reporter gene expression, penetrate deeper layers or target specific cell types with certain antibodies. We asked whether the ability to more reliably perform wholemount immunohistochemistry after dispase treatment could also be applied for the analysis of other markers (Figure S3) or tissues (Figure S4). Indeed, dispase treatment significantly improved the penetration of anti-laminin and anti-MRC1 immunostaining in cleared cerebellum wholemounts (Figure S4), and similarly reduced immunostaining of the outermost surface of the tissue (Figure S4). Nonetheless, antibodies that were once thought to be incompatible with tissue clearing may become effective after dispase treatment, which would provide additional opportunities to assess deep tissue structures with these markers. In summary, we demonstrate that depigmentation through hydrogen peroxide bleaching quenches endogenous fluorescence of GFP reporter transgenes, and that dispase treatment allows for immunolabeling of GFP reporters and reduces background staining at the sclera.

**Table 1:** List of primary and secondary antibodies

Antibody	Host/Clonality	Dilution	Catalog	Company	Dispase dependent
RedDot 2	NA	1:100	40,061–1	Biotium	No
SYTOX Orange	NA	1:15,000	S11368	Thermo Fisher Scientific	No
Anti-GFP	Goat/Polyclonal	1:50	600-101-215	Rockland Immunochemicals	Yes
Anti-Brn3a	Goat/Polyclonal	1:100	sc-31984	Santa Cruz Biotechnology	No
Anti-CD31	Rabbit/Polyclonal	1:50	ab28364	Abcam	No
Anti-Mrc1	Goat/Polyclonal	1:100	Af2535	R&D Systems	No
Anti-Laminin	Rabbit/Polyclonal	1:100	ab11575	Abcam	Yes
TO-PRO-3	NA	1:1000	T3605	Thermo Fisher Scientific	No
Anti-Cone Arrestin	Rabbit/Polyclonal	1:100	AB15282	Millipore	Yes
Donkey anti-rabbit IgG 647	Polyclonal	1:500	A-31573	Thermo Fisher Scientific	NA
Donkey anti-goat IgG 488	Polyclonal	1:500	A-11055	Thermo Fisher Scientific	NA
Donkey anti-Goat IgG 647	Polyclonal	1:500	A-21447	Thermo Fisher Scientific	NA

### InVision analysis of murine retinoblastoma

**Figure 4:**
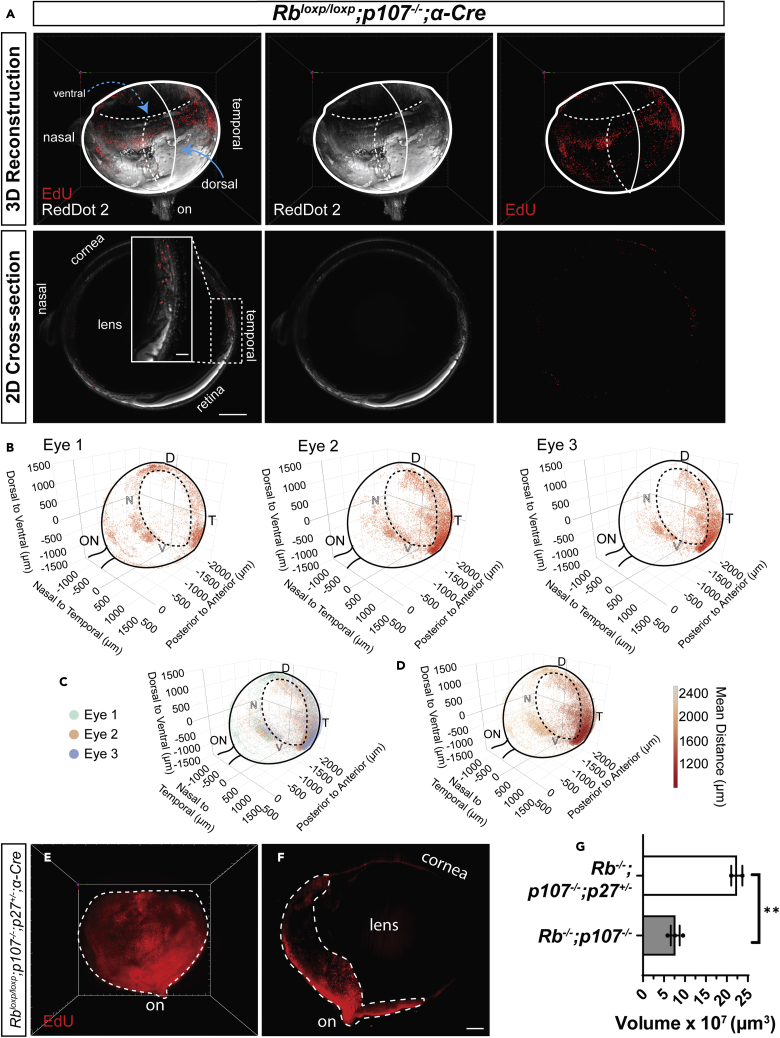
High-throughput 3D volumetric analysis in retinoblastoma models (A) 3D reconstruction and optical section of dispase treated, depigmented, cleared, EdU-labeled and RedDot2 nuclear-stained 1-month-old ⍺-*Cre; Rb*^loxp/*loxP*^; *p107*^−/−^ mouse eye. (B–D) (B and C) distribution and (D) density analysis of EdU-labeled cells in 1-month-old ⍺-*Cre; Rb*^loxp/*loxP*^; *p107*^−/−^ mouse eyes. The EdU-labeled cells are distributed in the periphery with a higher density in the anterior-ventral-temporal quadrant of the retina. Surviving, structurally intact retina can be distinguished morphologically from structurally disrupted, ectopically dividing EdU^+^ cells (A inset). Solid black lines represent the outline of the retinal cup and position of the optic nerve (ON). Dashed black lines represent the outline of the iris of the eye. (E and F) (E) 3D reconstruction and (F) optical section of an eye from a 6-month-old *α-Cre; Rb*^*loxp/loxP*^; *p107*^*−/−*^; *p27*^*+/−*^ mouse stained with EdU. The ectopically dividing peripheral retina (dashed line) displays a higher density of EdU^+^ cells in the central portion of the eye with reduced density closer to the periphery. (G) Comparison of the volume of EdU^+^ regions in the two retinoblastoma genotypes. Data are represented as means ± SEM, Unpaired ttest, ∗∗ denotes p < 0.01, p = 0.0032; n = 2 eyes (*α-Cre; Rb*^*loxp/loxP*^; *p107*^*−/−*^; *p27*^*+/−*^) and n = 3 eyes (⍺-*Cre; Rb*^loxp/*loxP*^; *p107*^−/−^ mouse eyes). Scale bars = 500 μm. D, dorsal; V, ventral; N, nasal; T, temporal. Solid lines in the (A) 3D reconstruction and (B) distribution plots represent the plane of the image toward the reader (out of the page), while dashed lines represent the plane of the image away from the reader (into the page).

Transgenic mouse models are useful to investigate tumorigenic mechanisms and pre-clinical therapeutics for retinoblastoma (RB), an intraocular tumor that develops from the neural retina. Here, we asked whether *InVision* could quantify early defects in a tumor-prone retina and/or advanced RB tumors. We utilized RB models in which conditional deletion of the *Rb1* tumor suppressor gene is combined with germline absence of the *Rb1* relative *p107* or the CDK inhibitor *p27* (Chen et al., 2004; Sangwan et al., 2012; Tables 2 and 3). Both models employ the *α-Cre* transgene, which is expressed early in embryonic retinal progenitors, deleting floxed alleles in most of the peripheral retina, except for a dorsal region and a smaller ventral portion, and sparing the entire central retina (Marquardt et al., 2001). In tumor-prone *Rb/p107*^*−/−*^ eyes, all differentiating cells exhibit abnormal division, causing apoptosis of most neurons, and while ectopically dividing amacrine and horizontal interneurons as well as Müller glia survive, nascent tumors arise from the amacrine lineage, generating RB in around half of *Rb/p107*^*−/−*^ eyes (Chen et al., 2004). Prior analysis of BrdU-incorporation in horizontal sections proximal to the optic nerve suggested that most ectopic division ceases by postnatal day (P) 30 (Chen et al., 2004). However, 3D analysis of EdU-incorporation in three one-month-old *α-Cre; Rb*^*loxp/loxP*^; *p107*^*−/−*^ eyes exposed ectopic division in every case, particularly in the temporal region (Figures 4A and 4B and Video S3). Nearest neighbor analysis revealed that the anterior-ventral-temporal region exhibited most EdU-incorporation, particularly in eyes #2 and #3 (Figures 4C and 4D). These data highlight the comprehensive architectural insight afforded by 3D *vs*. 2D analysis. The majority of EdU^+^ cells in the young retina represent ectopic division that will eventually cease, contrasting the extensive, de-regulated division in a mature tumor. Indeed, 3D imaging of tumors in 6-month-old *α-Cre; Rb*^*loxp/loxP*^; *p107*^*−/−*^; *p27*^*+/−*^ eyes confirmed a much larger volume of EdU^+^ cells than in 1-month old *α-Cre; Rb*^*loxp/loxP*^; *p107*^*−/−*^ eyes (22.37 ± 1.35 × 10^7^μm^3^ vs 7.68 ± 1.06 × 10^7^μm^3^, p = 0.0032; Figures 4E–4G and Video S3). These results demonstrate the utility of *InVision* to track quantitatively and qualitatively both early- and late-stage murine RB.

**Table 2:** Mouse lines used for respective analysis and age of mice

Genotype	Analysis	Age
*Pigmented* C57BL/6J	Vasculature analysis	1 month
*Pigmented Ccdc-136*^*GFP/GFP*^	Establishment of the InVision protocol	1 month
*Pigmented Rb*^*loxp/loxP*^; *p107*^−/−^; *α-Cre* (Chen et al., 2004)	EdU analysis	1 month
*Pigmented Rb*^*loxp/loxP*^; *p107*^*−/−*^; *p27*^*+/−*^; *α-Cre* (Chen et al., 2004)	EdU analysis	6 months
*Pigmented Ndp*^−/*Y*^	Vasculature analysis	1 month
*Nrl-GFP*	Donor photoreceptors for transplantation	P3-P5
*Albino ROSA*^*mT/mG*^	Establishment of the InVision protocol	1 month
*Albino ROSA*^*mT/mG*^	Neurodegeneration	1, 3 and 6 months
*Albino ROSA*^*mT/mG*^	Transplantation recipient	2-3 months
*Albino Nrl*^*−/−*^;*ROSA*^*mT/mG*^	Neurodegeneration	1, 3 and 6 months
*Albino Nrl*^*−/−*^;*ROSA*^*mT/mG*^	Transplantation recipient	2-3 months

**Table 3:** PCR genotyping primers for respective mouse lines.

Genotype	Target	Forward primer	Reverse primer
*Pigmented* C57BL/6J	NA	NA	NA
*Pigmented Ccdc-136*^*GFP/GFP*^	Wild type	CCGTGGTGGGGGTTGAATCCAA	TGGCAAAGTCATGAAGGGACCACA
*Pigmented Ccdc-136*^*GFP/GFP*^	Mutant	CACATGAAGCAGCACGACTT	TGCTCAGGTAGTGGTTGTCG
*Pigmented Rb*^*loxp/loxP*^; *p107*^−/−^; *α-Cre*	*Rb floxed*	CGC CGC ATA ACC AGT GAA AC	CTC AAG AGC TCA GAC TCA TGG
*Pigmented Rb*^*loxp/loxP*^; *p107*^−/−^; *α-Cre*	*p107*^−/−^	TCG TGA GCG GAT AGA AAG	GTG TCC AGC AGA AGT TA
*Pigmented Rb*^*loxp/loxP*^; *p107*^−/−^; *α-Cre*	*Cre*	ATG TCC AAT TTA CTG ACCG	CGC CGC ATA ACC AGT GAA AC
*Pigmented Rb*^*loxp/loxP*^; *p107*^*−/−*^; *p27*^*+/−*^; *α-Cre*{	*p27*^*+/+*^	GCC TGG CTC TGC TCC ATT TGA C	CTC TCC ACC TCC TGC CAT TC
*Pigmented Rb*^*loxp/loxP*^; *p107*^*−/−*^; *p27*^*+/−*^; *α-Cre*	*p27*^*−/−*^	CCT TCT ATG GCC TTC TTG ACG	TGG AAC CCT GTG CCA TCT CTA T
*Pigmented Ndp*^−/*Y*^	Wild Type	NA	CAGGGAGAGCATAGAAATGG
*Pigmented Ndp*^−/*Y*^	Mutant	TCTGATTTCATTCCAGCTGTG	CCCTAGGAATGCTCGTCAAGA
*Nrl-GFP*	Wild type	GTGTTCCTTGGCTGGAAAGA	CTGTTCACTGTGGGCTTTCA
*Nrl-GFP*	Mutant	CACATGAAGCAGCACGACTT	TGCTCAGGTAGTGGTTGTCG
*Albino ROSA*^*mT/mG*^	Wild type	GGA GCG GGA GAA ATG GAT ATG	NA
*Albino ROSA*^*mT/mG*^	Mutant	GCG AAG AGT TTG TCC TCA ACC	NA
*Albino ROSA*^*mT/mG*^	Common	NA	AAA GTC GCT CTG AGT TGT TAT
*Albino Nrl*^*−/−*^;*ROSA*^*mT/mG*^	*Nrl* wild type	GTGTTCCTTGGCTGGAAAGA	CTGTTCACTGTGGGCTTTCA
*Albino Nrl*^*−/−*^;*ROSA*^*mT/mG*^	*Nrl* mutant	TGAATACAGGGACGACACCA	GTTCTAATTCCATCAGAAGCTGAC
*Albino Nrl*^*−/−*^;*ROSA*^*mT/mG*^	*ROSA* wild type	GGA GCG GGA GAA ATG GAT ATG	NA
*Albino Nrl*^*−/−*^;*ROSA*^*mT/mG*^	*ROSA* mutant	GCG AAG AGT TTG TCC TCA ACC	NA
*Albino Nrl*^*−/−*^;*ROSA*^*mT/mG*^	*ROSA* Common	NA	AAA GTC GCT CTG AGT TGT TAT

Video S3. 3D reconstruction of retinal tumors, related to Figure 4 3D reconstruction of 1-month-old ⍺-*Cre*; *Rb*^loxp/*lox*^^*p*^; *p107*^−/−^ and 6-month-old ⍺-*Cre*; *Rb*^loxp/*lox*^^*p*^; *p107*^−/−^; *p27*^+/−^ mouse eyes with EdU-labelled cells. EdU (red), RedDot2 (white).

### InVision analysis of retinal vasculature pathology in Norrie disease eyes

**Figure 5:**
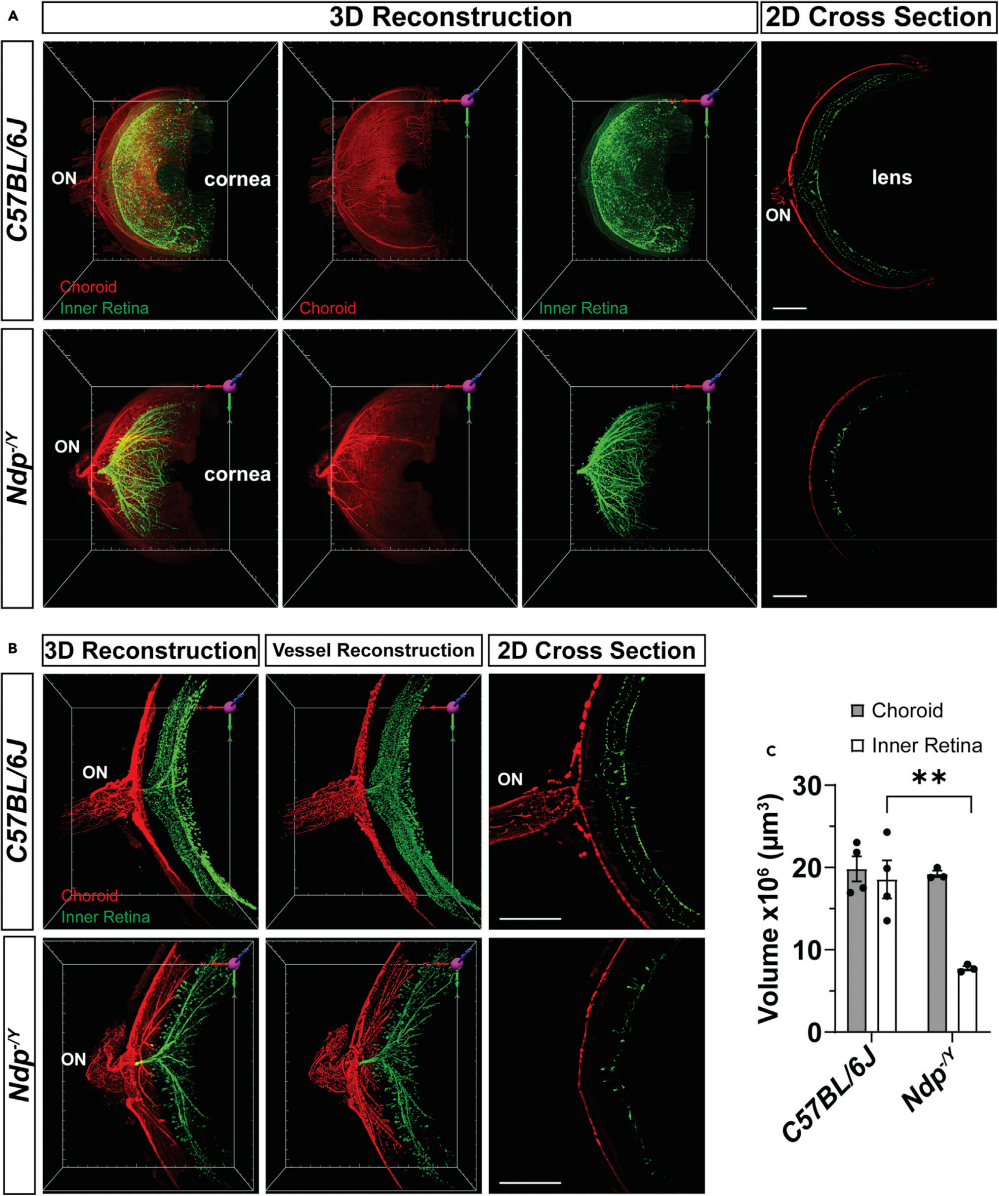
3D volumetric analysis of wildtype and *Ndp*^*-/Y*^retinal and choroidal vasculature in cleared eyes (A) 3D reconstruction and 2D optical cross sections of dispase treated, depigmented, cleared eyes from C57BL/6J and *Ndp*^*-/Y*^ stained with anti-CD31 antibodies to detect endothelial cells. The choroidal (red) has been manually segmented from the inner retinal (green) vessels and pseudo-colored. (B and C) (B) High magnification 3D reconstruction and 2D optical cross sections of CD31 stained retinal vessels in 1-month old C57BL/6J and *Ndp*^−/*Y*^ retinas. Images were taken as ~1mm area centered at the optic nerve. The choroidal (red) has been manually segmented from the inner retinal (green) vessels and pseudo-colored. Hypovascularization of the inner retina is evident at (A) low and (B) high magnifications. Semi-automatically derived 3D vessel reconstruction of the CD31 stain accurately models the fluorescent CD31 signal. The 3D vessel construction allows for the (C) volumetric comparison of choroidal and inner retinal vessels in both C57BL/6J and *Ndp*^−/*Y*^ retinas. Scale bars = 500 μm. Data are represented as means ± SEM, two-way ANOVA with Tukey's post-hoc multiple comparisons test, ∗∗ denotes p < 0.01, p = 0.0043; n= 4 eyes (C57BL/6J) and 3 eyes (*Ndp*^−/*Y*^) with vessel (choroid and inner) and genotype (C57BL/6J and *Ndp*^−/*Y*^) as factors.

Pathological vascularization contributes to vision loss in a variety of eye diseases, including retinopathy of prematurity, diabetic retinopathy, and choroidal neovascularization in age-related macular degeneration (Campochiaro, 2013). Investigations into the underlying regulatory mechanisms of normal and pathological angiogenesis are frequently carried using the mouse eye. Norrin, the secreted protein product encoded by the *Norrie Disease Protein* (*Ndp*) gene, is necessary for retinal vascular development and maintenance in humans and mice (Ye et al., 2010). *Ndp*^*-/Y*^ mouse retinas exhibit hypovascularization that mimics that of human Norrie disease (Ye et al., 2009). To evaluate the applicability of *InVision* toward the analysis of vascular defects, we imaged 1-month-old *Ndp*^*-/Y*^ and wildtype mouse retinas and used Imaris to segment the choroidal and retinal blood vessels (Figure 5). Evaluation of C57BL/6J eyes revealed robust and detailed CD31^+^ signal in the retinal and choroidal vasculature (Figure 5). Consistent with previous reports, *Ndp*^*-/Y*^ retinas exhibited severe hypovascularization and aberrant clusters of endothelial cells that penetrated the retina from the vitreal surface (Ye et al., 2009) (Figures 5A and S5). Semi-automatic quantification of vascular volume revealed that *Ndp*^*-/Y*^ retinal vessels surrounding the optic nerve head were significantly reduced (7.75 ± 0.266 × 10^6^μm^3^) relative to wild type littermates (18.6 ± 2.31 × 10^6^μm^3^, p = 0.0043). In contrast, *Ndp*^*-/Y*^ choroidal vessel volume was not significantly different from wildtype (19.8 ± 1.53 × 10^6^μm^3^ and 19.2 ± 0.387 × 10^6^μm^3^, respectively; p = 0.9930; Figures 5B and 5C), indicating that the Norrie disease phenotype is specific to retinal and not choroidal vasculature.

### Evaluating retinal degeneration using InVision analysis

**Figure 6:**
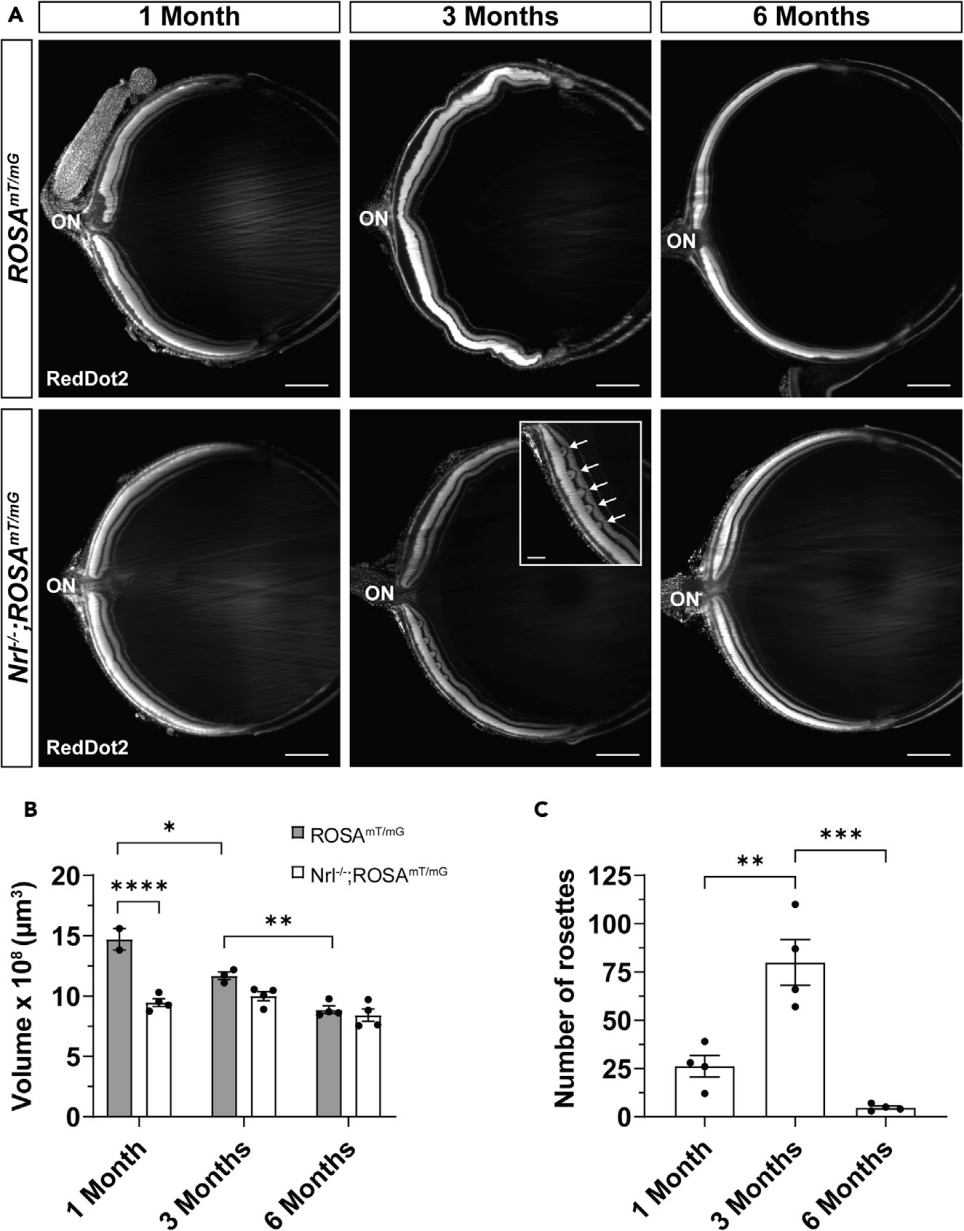
3D volumetric analysis of neurodegenerative phenotypes in cleared mouse eyes (A) Optical sections of cleared eyes from wild type albino *ROSA*^*mT/mG*^ (top row) and *Nrl*^*−/−*^;*ROSA*^*mT/mG*^ (bottom row) mice at 1, 3, and 6 months of age stained with nuclear RedDot2. Rosettes (inset, arrows) in the ONL of the *Nrl*^*−/−*^;*ROSA*^*mT/mG*^ retina are most frequent at 3 months. (B) ONL volume in *ROSA*^*mT/mG*^ (n = 2, 3, 4 eyes) and *Nrl*^*−/−*^;*ROSA*^*mT/mG*^ (n = 4, 4, 4 eyes) retinas at 1, 3, and 6 months, respectively. (C) Number of rosettes in *Nrl*^*−/−*^;*ROSA*^*mT/mG*^ retinas (n = 4, 4, 4 eyes at 1, 3, and 6 months, respectively). Scale bars = 500 μm for A and 150 μm for A, inset. Data are represented as means ± SEM. ∗p < 0.05, ∗∗p < 0.01, ∗∗∗p < 0.001, ∗∗∗∗p < 0.0001. Two-way ANOVA with Tukey’s post-hoc multiple comparisons test for B with age (1, 3, and 6 months) and genotype (*ROSA*^*mT/mG*^ and *Nrl*^*−/−*^;*ROSA*^*mT/mG*^) as factors. One-way ANOVA with Tukey’s post-hoc multiple comparisons test for C with age (1, 3, and 6 months) as the factor. ON, optic nerve.

Photoreceptor degeneration can be modeled in a variety of mouse models and is typically characterized by a reduction in the thickness of the ONL and, in some models, undulations of the ONL, referred to as rosettes (Stuck et al., 2012). Monitoring these abnormalities in the mouse retina is a useful measure to track the impact of therapeutic interventions on disease progression (Cideciyan et al., 2013; Martinez-de-la-Casa et al., 2012; Talcott et al., 2011). We used *InVision* analysis to characterize the progressive phenotypic changes in retinal architecture of the *Nrl*^*−/−*^ retina (Figure 6), a mouse model of aberrant photoreceptor lineage specification and degeneration (Mears et al., 2001). Consistent with previous reports (Roger et al., 2012), we observed a significant decrease of the ONL volume by 1 month in *Nrl*^*−/−*^ (9.47 ± 0.32 × 10^8^μm^3^) retinas, compared to wildtype controls (14.7 ± 0.89 × 10^8^μm^3^;p < 0.0001), before stabilizing at 6 months (Figure 6B). Meanwhile, the ONL of wildtype retinas continued to thin at 3 months (11.7 ± 0.31 × 10^8^μm^3^; 1 month vs 3 months p = 0.0128) and 6 months (8.88 ± 0.30 × 10^8^μm^3^; 3 months vs 6 months p = 0.0051). The number of rosettes in the *Nrl*^*−/−*^ retina was significantly increased between 1 (26.3 ± 5.5) and 3 months (80.0 ± 11.8; p = 0.0018) followed by a significant rapid decline by about 6 months (4.75 ± 0.85; p = 0.0002; Figure 6C). Collectively, these data demonstrate how the *InVision* protocol permits a corroborative analysis of layer volume and a pathological phenotype of the ONL over time.

### InVision imaging and analysis of transplanted retinal photoreceptors

Photoreceptor degeneration causes permanent, untreatable vision loss (Wright et al., 2010); hence, there has been considerable effort to investigate the feasibility of photoreceptor replacement to restore light sensitivity to the retina. Indeed, transplantation of GFP^+^-photoreceptor precursor cells results in the appearance of GFP^+^-photoreceptors that reside in the host retina and is reported to improve light sensitivity and vision in blind mouse models (Barber et al., 2013; Barnea-Cramer et al., 2016; MacLaren et al., 2006; Pearson et al., 2012; Warre-Cornish et al., 2014). However, recent reports show that GFP^+^-donor photoreceptors almost never “integrate” into the host retina and instead the GFP labeling detected in the host retina arises through transfer of fluorescent reporters between donor and host photoreceptors, in a process termed material transfer (MT) (Decembrini et al., 2017; Ortin-Martinez et al., 2017; Pearson et al., 2016; Santos-Ferreira et al., 2016; Singh et al., 2016). The mechanism of MT is unknown, but this process may explain photoreceptor-transplant-induced functional rescue.

**Figure 7:**
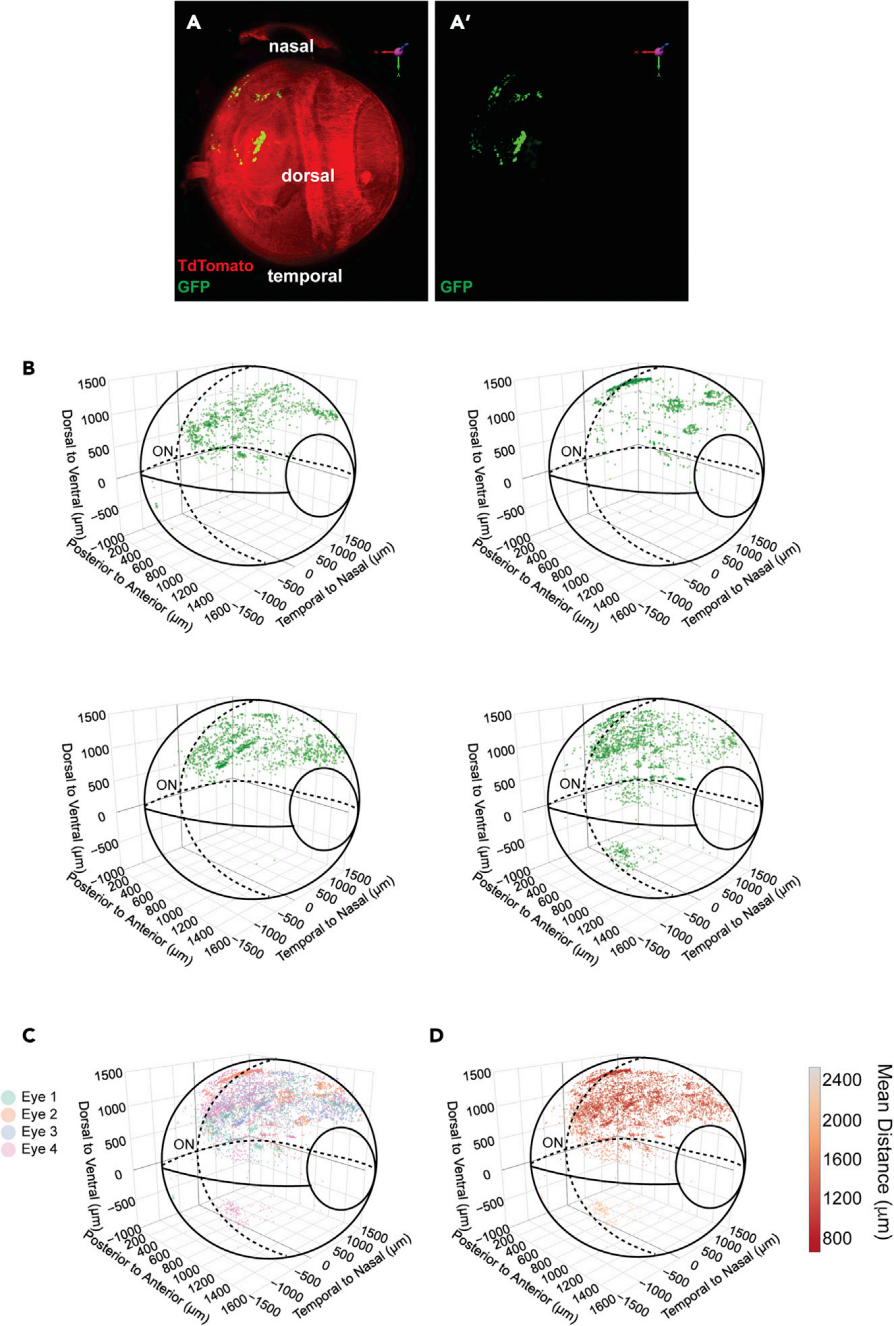
High-throughput analysis of subretinal photoreceptor transplants in cleared eyes (A) 3D reconstruction of an intact adult albino *ROSA*^*mT/mG*^ eye 3 weeks after subretinal transplantation with *Nrl*-GFP retinal dissociates. The location and distribution of GFP^+^ cells within the eye is detectable. (B and C) (B) The distribution of the transplanted GFP^+^ cells in four different eyes and the subsequent (C) overlay of the distributions. GFP^+^ cells are predominantly located in the superior nasal quadrant of the globe. (D) The density distribution of (C). Sub-retinal transplants have a significant degree of animal to animal variability but are (C) overall enriched in the superior nasal quadrant with a (D) greater density (red) of cells in the superior part of the eye and reduced density (orange-yellow) in the inferior most part of the eye. Solid lines in the (B-D) distribution plots represent the plane of the image toward the reader (out of the page), while dashed lines represent the plane of the image away from the reader (into the page). ON, optic nerve.

**Figure 8:**
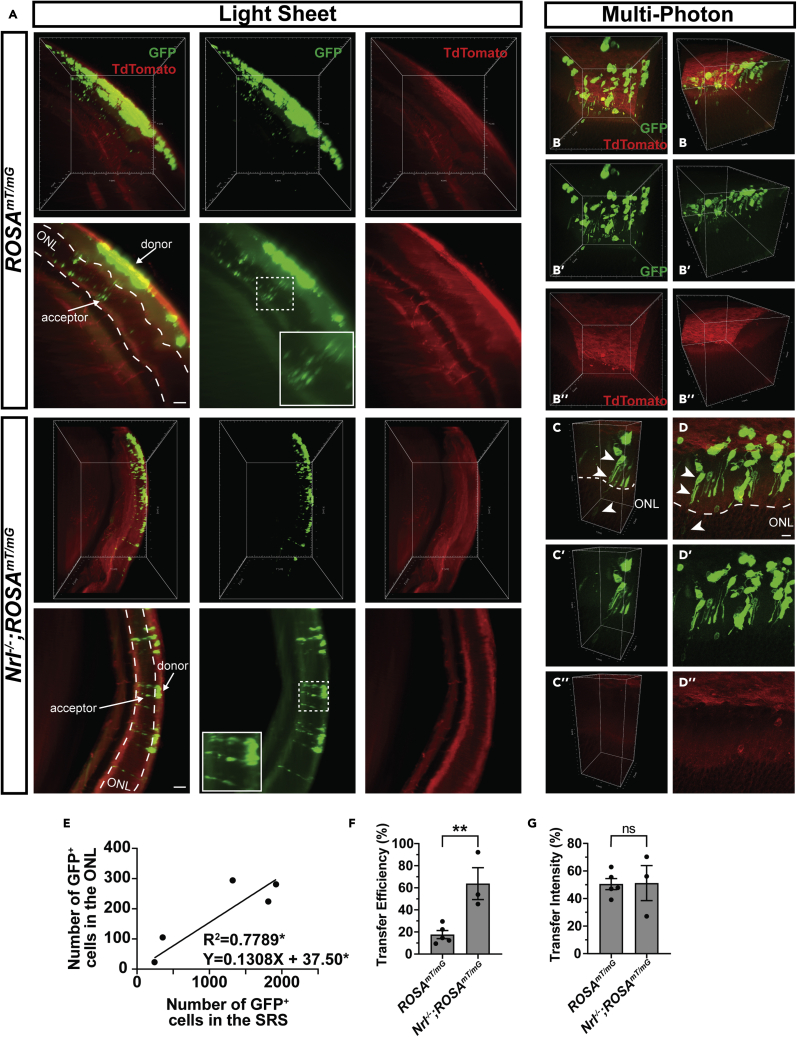
Single cell resolution of acceptor cells in transplanted retinas (A) 3D reconstruction and optical sections of a magnified region of *ROSA*^*mT/mG*^ and *Nrl*^*−/−*^;*ROSA*^*mT/mG*^ transplanted retinas. Acceptor cells are detectable at a single cell resolution. (B) 3D reconstruction of a *ROSA*^*mT/mG*^ recipient retina using MP imaging. (C and D) (C) A cropped region and (D) optical section of the 3D reconstructed MP image in (B). Processes from donor photoreceptors (top arrow) appear to be making contact (middle arrow) with the host photoreceptor in the ONL (bottom arrow). (E) Linear correlation between the number of GFP^+^ donor cells in the SRS and acceptor cells in the ONL in *ROSA*^*mT/mG*^ recipient retinas. Pearson r correlation, p = 0.0474, R^2^ = 0.78, n = 5 eyes. (F and G) Comparison of the transfer efficiency and transfer intensity between *ROSA*^*mT/mG*^ and *Nrl*^*−/−*^;*ROSA*^*mT/mG*^ recipients. Data are represented as means ± SEM. ∗∗ denotes p < 0.01. Unpaired t-tests, p = 0.0073 and p = 0.9501 respectively, n = 5 (*ROSA*^*mT/mG*^) and n = 3 (*Nrl*^*−/−*^;*ROSA*^*mT/mG*^) eyes. ONL, outer nuclear layer. Scale bars = 50 μm for A, 10 μm for D.

To investigate the applicability of *InVision* to characterize cell transplantation and MT we imaged cleared adult albino *ROSA*^*mT/mG*^ recipient eyes 21 days after transplantation with GFP^+^-photoreceptor precursors (Figure 7A). Lower-magnification LSFM imaging of *InVision*-treated eyes revealed that GFP^+^-donor cells were readily detectable and while they were dispersed preferentially in the region where they were injected, the superior nasal quadrant of the eye, there was still a previously unappreciated degree of variability in their distribution (Figures 7A–7D). The ease of detecting transplanted cells in whole eye imaging contrasts with the more traditional analysis in 2D tissue sections, which typically requires extensive sampling across the entire eye to locate donor cells. Next, using a 20X, solvent-compatible lens on the light sheet microscope, we were able to image single GFP^+^-acceptor photoreceptors (Figure 8A and Video S4), which represent host cells that acquire GFP through MT from donor cells. Although highly informative at lower magnifications, LSFM has a laser sheet depth limitation of approximately 5 μm, necessitating an alternative imaging modality for high-magnification analysis. Therefore, we tested the feasibility of imaging *InVision* eyes using MP microscopy, which has the added advantage of high-penetrating laser excitation while maintaining high z resolution. Moving the specimen from LSFM to MP imaging dramatically improved the interpretation of cell morphology and z distribution, revealing donor cells consistently located in close proximity apical to acceptor cells (Figure 8B andVideo S5). Donor cells also exhibited processes that extended toward acceptor cell inner segments (Figure 8B andVideo S6). Collectively, these data indicate that *InVision* can be used in multimodal microscopic imaging within a single sample, thus combining the advantages of whole organ and single-cell resolution microscopy.

Video S4. LSFM-acquired, 3D reconstructed image of a transplanted eye, related to Figure 8 3D reconstruction with LSFM of an albino *ROSA*^*mT/mG*^
mouse eye (red) 21 days after transplantation with *Nrl*-*GFP* donor cells (green). TOPRO (blue).

Video S5. MP-acquired, 3D reconstructed image of a transplanted eye, related to Figure 8 3D reconstruction using MP imaging of a region of a transplanted albino *ROSA*^*mT/mG*^
mouse eye (red) showing the location of *Nrl*-*GFP* donor cells (green).

Video S6. Cropped region of Video S5, related to Figure 8 3D reconstruction using MP imaging of a cropped region from Video S5 of an albino *ROSA*^*mT/mG*^
mouse eye (red) transplanted with *Nrl*-*GFP* donor cells (green).

Next, we investigated whether we could use the *InVision* approach to quantify the degree of MT. However, because of the variability in donor cell number between transplanted eyes (Figure 8C) we decided to compare donor and acceptor cell numbers between recipient eyes. Imaging a ∼1 mm^3^ region of the intact transplant and cell quantification revealed that the number of GFP^+^-donor cells in the subretinal space (SRS) was linearly correlated with the number of GFP^+^-acceptor photoreceptors located in the ONL (R^2^ = 0.7789, p = 0.0474; Figure 8C). This observation indicates that the degree of transfer is dependent on the number of donor cells. Thus, given the ability to more accurately sample transplanted regions for donor and acceptor cell number, we compared differences in MT between wildtype and *Nrl*^*−/−*^ recipients, which have previously been shown to have a higher amount of MT (Ortin-Martinez et al., 2017; Santos-Ferreira et al., 2015), using a new parameter, transfer efficiency (number of GFP^+^-acceptor cells in the host ONL/number of GFP^+^-donor cells in the SRS). *InVision*-prepared, *Nrl*^*−/−*^ recipient retinas exhibited a significant increase in GFP transfer (63.8 ± 14.4%) compared to their wild type counterparts (17.7 ± 3.7%; p = 0.0073; Figure 8D), which is consistent with work that used traditional cryosectioning to quantify GFP^+^-acceptor cells in *Nrl*^*−/−*^ transplanted eyes (Ortin-Martinez et al., 2017; Santos-Ferreira et al., 2015). Comparison of GFP intensity in donor and acceptor populations revealed that the relative intensity of GFP in acceptor cells is weaker relative to donor cells between both genotypes (intensity of acceptor cell GFP/intensity of donor cell GFP, wild type: 50.5 ± 4.0% and *Nrl*^*−/−*^: 51.2 ± 12.7%), which is consistent with previous work (Gasparini et al., 2019). However, the relative GFP signal intensity was not significantly different between the two genotypes (p = 0.95; Figure 8E), suggesting that better accumulation of GFP in acceptor cells does not explain why MT is more efficient in *Nrl*^*−/−*^ recipients.

**Figure 9:**
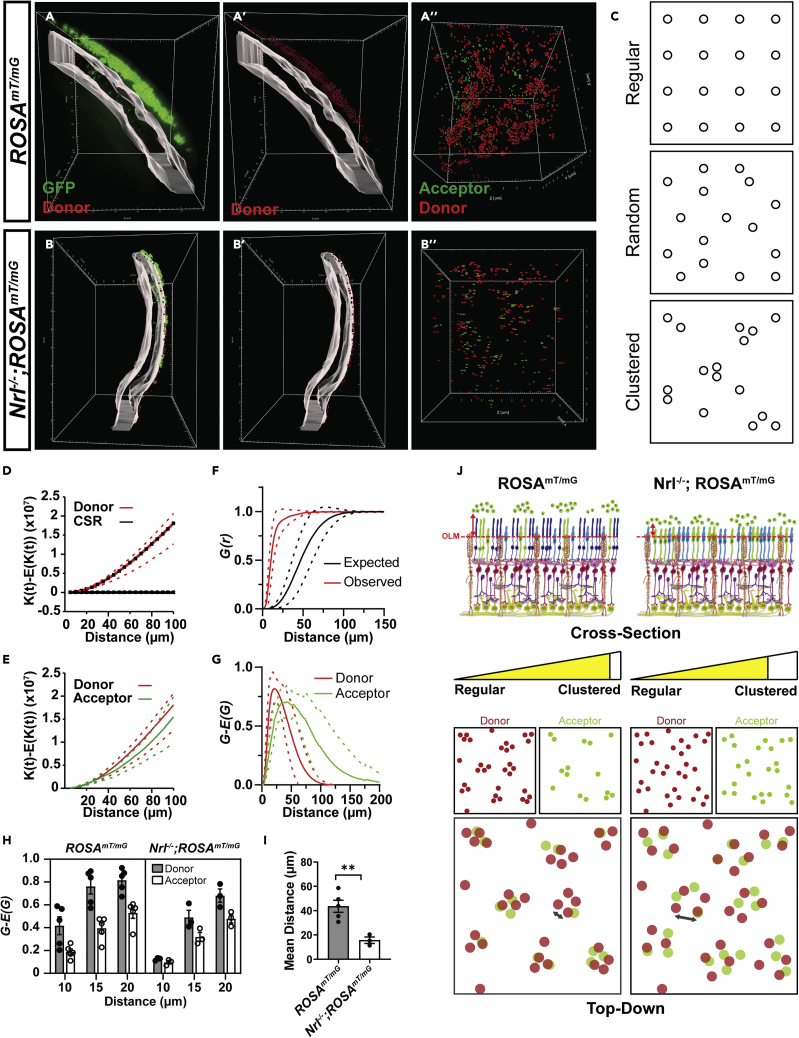
3D Spatial analysis of intact cleared eyes reveals genotype specific differences mediating material exchange (A and B) Spot analysis of GFP^+^ donor and acceptor cells within transplanted (A) *ROSA*^*mT/mG*^ and (B) *Nrl*^*−/−*^; *ROSA*^*mT/mG*^ recipient retinas. GFP^+^ somas present within the volume of the ONL (gray) were classified as acceptor cells while GFP^+^ somas present outside the ONL, within the SRS, were classified as donor cells. The center of GFP^+^ (A′, A″) donor (red) and (A″ and B″) acceptor (green) somas are represented as spots within the volume of the transplanted region. Donor cells in (A′) *ROSA*^*mT/mG*^ recipient retinas are located further away from the apical most edge of the ONL volume compared to donor cells in (B′) *Nrl*^*−/−*^;*ROSA*^*mT/mG*^. Both donor and acceptor cell somas appear to overlap along the plane normal to the ONL in both (A″) *ROSA*^*mT/mG*^ and (B″) *Nrl*^*−/−*^;*ROSA*^*mT/mG*^ recipient retinas. (C) Examples of regular, random and clustered point pattern distributions. (D) Comparison of the difference in observed and expected K functions for donor cell populations in *ROSA*^*mT/mG*^ recipients and complete spatial randomness (CSR). That the difference between observed and expected K functions was greater than the CSR curve suggests clustered distributions. (E) Comparison of the difference in observed and expected K functions for donor and acceptor cell populations in *ROSA*^*mT/mG*^ recipients. (F) Plots of the nearest neighbor distance G-function for observed and expected donor cell populations in *ROSA*^*mT/mG*^ recipients. That the observed G functions are left shifted compared to the expected suggests a clustered population. (G) Comparison of the difference in observed and expected G functions for donor and acceptor cell populations in *ROSA*^*mT/mG*^ recipients. (H) Comparison of the difference in observed and expected G functions for donor and acceptor cell populations in *ROSA*^*mT/mG*^ and *Nrl*^*−/−*^;*ROSA*^*mT/mG*^ recipients, at 10, 15 and 20 μm distances. (I) Comparison of the mean shortest distance of donor cells to the OLM in *ROSA*^*mT/mG*^ and *Nrl*^*−/−*^;*ROSA*^*mT/mG*^ recipient retinas. (J) Summary schematic of the differences in 3D distribution patterns of donor and acceptor cells in *ROSA*^*mT/mG*^ and *Nrl*^*−/−*^;*ROSA*^*mT/mG*^ recipient retinas. Top (cross sectional view of the transplanted region): Donor cells in *Nrl*^*−/−*^;*ROSA*^*mT/mG*^ recipient retinas exhibit a reduced distance (red arrow) to the OLM compared to donor cells in *ROSA*^*mT/mG*^ recipient retinas. Bottom (top-down view of the transplanted region): Both donor (red circles) and acceptor (green circles) cell populations exhibit less clustering (yellow meter) in *Nrl*^*−/−*^;*ROSA*^*mT/mG*^ recipients compared to *ROSA*^*mT/mG*^ recipients. Gray arrows, in overlay panels of donor and acceptor populations for each recipient genotype, highlight the difference in distance between donor (red circles) or between acceptor (green circles) cells in each recipient genotype. Data are represented as means ± SEM. ∗∗denotes p < 0.01. Unpaired t-test for I, p = 0.0067, n = 5 (*ROSA^mT/mG^*) and n = 3 (*Nrl^-/-^*;*ROSA^mT/mG^*) eyes. The dashed lines in D, E, F and G represent the 95% confidence intervals.

The traditional 2D sectioning and imaging approach used to analyze MT suffers from limitations in the ability to fully query the physical relationship between donor and acceptor photoreceptors in a global transplant context. For example, donor and host cells frequently reside in different cutting planes, making it difficult to interpret how these cells interact based on the analysis of physical tissue sections. We reasoned that by exploiting whole-tissue imaging and 3D spatial distribution analysis of donor and acceptor cells, we could gain insight about the mechanism of MT. For example, if MT is mediated via long-range modes of cellular communication, such as the exchange of extracellular vesicles, one might expect the distribution of donor and acceptor cells to not overlap. If, however, MT is mediated by short range interactions, the distribution pattern of donor and acceptor cells should overlap. We noted, through visual inspection of 3D reconstructed transplants, that acceptor cells were consistently located basal to one or more donor cells in the subretinal space, normal to the outer limiting membrane (OLM), an apical network of junctional complexes between photoreceptors and glial cells. In other words, acceptor and donor cells were aligned in 3D space (Figures 9A and 9B). Thus, we performed an in-depth quantitative analysis of the 3D spatial relationship between populations of donor and acceptor cells. Using Ripley’s K Function (Hansson et al., 2013), we first statistically evaluated whether the distribution of donor and acceptor photoreceptors in wild-type and *Nrl*^*−/−*^ recipients followed a clustered, completely random (CSR) or regularly spaced distribution (Figure 9C). Consistent with images of the *en face* view of donor and acceptor cell distributions (Figure 9A” and B″), both donor and acceptor populations in wild type (p < 0.001 and p < 0.001, respectively; Figures 9D and S6A) and *Nrl*^*−/−*^ (p < 0.001 and p <0.001, respectively; Figures S6B and S6C), were significantly different from distributions that follow CSR. That the K-functions are above the line for the CSR also indicates a clustered type distribution, as opposed to a regularly spaced or dispersed distribution (Figures 9D and S6A–S6C). Moreover, for both wild type and *Nrl*^*−/−*^ recipients, the distribution of donor and acceptor cells was not statistically different (p = 0.7934 and p = 0.4649, respectively; Figures 9E and S6D). Together, these data show that acceptor cells are spatially correlated on the position and distribution of donor photoreceptors. The alignment in the distribution patterns also suggests that MT is mediated by a short-range interaction, which is consistent with the observation that donor cells extended neurite-like processes that appear to contact with acceptor cells (Figure 8B).

Although donor and acceptor cells exhibited a clustered distribution pattern in both types of recipients, further analysis revealed some genotype-specific differences that correlated with differences in MT efficiency. By evaluating the difference between the observed and expected nearest neighbor distance function (G-E[G(r)]) at an r (distance) value of 10, 15, and 20 μm, we found that donor and acceptor cells were relatively less clustered in *Nrl*^*−/−*^ recipients compared to wild type counterparts (p = 0.0122; Figures 9F–9H and S6E–S6G). In addition, donor cells also exhibited a significantly shorter Euclidian distance to the OLM in *Nrl*^*−/−*^ recipients (15.7 ± 2.62 μm) compared to wild type (43.6 ± 4.96 μm) recipient retinas (p = 0.0067; Figure 9I). *Nrl*^*−/−*^ photoreceptors have shortened outer segments (Stuck et al., 2012), which could account for the increased donor cell proximity to the OLM and subsequent enhancement of transfer. Consistent with this hypothesis, we saw a similar enhancement of transfer in *Prph2*^*−/−*^ recipients, which also display outer segment defects (Farjo et al., 2007; Sanyal and Jansen, 1981) (Figure S6H). Collectively, these observations suggest that MT occurs via a short-range, contact-dependent mechanism when there is close positioning between donor and acceptor cells. Moreover, the enhancement of MT in *Nrl*^*−/−*^ recipients may be explained by reduced donor cell clustering and closer donor cell proximity to the host OLM which increases the probability for exchange with acceptor cells (Figure 9J).

## Discussion

We demonstrate the broad applicability of the *InVision* protocol to the imaging of several physiological processes studied using the ocular system as model. Although our method and applications are optimized for the rodent eye, combining depigmentation and permeabilization steps with optical clearing will permit this approach to be tested in other heterogeneous, pigmented, or collagen-enriched tissues. Recently, other groups have published modified versions of clearing protocols, such as seeDB (Hohberger et al., 2017), ethanol-ECi (EyeCi) (Henning et al., 2019), iDISCO (EyeDISCO) (Vigouroux et al., 2020), PACT (Prahst et al., 2020) and CUBIC (Ye et al., 2020). These studies encompass a wide variety of applications and imaging modalities. For example, although (Ye et al. 2020) utilized a CUBIC based protocol, the application toward an LSFM was not demonstrated. Some of these methods (Henning et al., 2019; Vigouroux et al., 2020; Ye et al., 2020) address the issue of pigmentation in a similar manner to our protocol. However, our attempt to resolve immunogenicity of intact eyes offers a novel approach over existing methods which involve antibody injection directly into the eye (Henning et al., 2019; Ye et al., 2020) or an incision of the cornea (Vigouroux et al., 2020). Enzymatic digestion with dispase may have its own advantages and disadvantages over previously described methods, however, research groups only stand to benefit from a myriad of available approaches that can be tailored to their specific applications. Another advantage of this technique is that cleared tissue may be serially imaged using light sheet, MP and confocal microscopes, and transfer between these imaging modalities is easily achieved using custom made 3D printed ocular sample holders. Moreover, after clearing, tissues may still be sucrose dehydrated before embedding in OCT compound (Sakura Finetek, #4583) for traditional 2D sectioning (Nojima et al., 2017; Susaki et al., 2014, 2015) (Figure S2B). Using traditional 2D sectioning to sample the entire adult rodent eye requires the analysis of ∼150 sections (eye measuring ∼3 mm diameter, ∼ 20 μm thick sections). Additionally, imaging sections on an epifluorescence or confocal microscope requires a significant time investment to image and analyze each section. In contrast, the LSFM is capable of imaging an entire 3 mm diameter eyeball at ∼5 μm Z axial resolution and 2x sampling at roughly 10 minutes/channel. Ultimately, *InVision* reduces tissue processing and imaging times and maintains the intact 3D ocular structure for quicker multimodal imaging, rendering, and analysis. Notably, modified versions of the CUBIC protocol have been recently developed (Murakami et al., 2018; Tainaka et al., 2018) since the beginning of our study and further work aims to incorporate and determine whether these more recent CUBIC protocols can improve on the *InVision* pipeline.

Ocular tumor models exhibit the challenge of imaging a structure that does not conform to a predictable size and shape. The ⍺-*Cre; Rb*^loxp/*loxP*^; *p107*^−/−^ mouse is no exception, necessitating an improvement in the speed by which one can evaluate ectopic proliferation and tumor status. We demonstrate that EdU-Click-iT chemistry is highly compatible with *InVision* and provides robust and complete distribution data of proliferative cells. Extending this work to include detailed tumor markers will greatly facilitate high-throughput imaging for interventional studies, such as screening the effects of intraocular compounds on tumor proliferation.

The detailed and complicated microanatomy of ocular vasculature makes it a particularly difficult tissue to image and render using serial cryosections. We used the *InVision* protocol and anti-CD31 immunohistochemistry to evaluate vasculature defects in the pigmented, *Norrie disease protein* knockout (*Ndp*^−/*Y*^) mouse. Differences in the inner retinal vessel network in adult *Ndp*^*-/Y*^ compared to wildtype eyes were easily interpreted using an intact mouse eye approach. Although we limited our analysis to a region around the optic nerve, imaging of whole-eye vasculature at sufficient resolution for segmentation can be achieved by increasing imaging time and file sizes to include all ocular anatomy. That LSFM imaging of intact vascular structures provides significant advantages over traditional confocal imaging of retinal wholemounts is described previously (Prahst et al., 2020; Singh et al., 2017). However, both (Singh et al., 2017) and (Prahst et al., 2020) performed their imaging and analysis on dissected retinal cups. In contrast, the *InVision* approach allows for vasculature analysis of the completely intact eye providing the opportunity to effectively study vasculature development of the choroid with minimal tissue manipulation.

We also demonstrate the deployment of our method for the analysis of neurodegeneration in *Nrl*^*−/−*^ retinas. *InVision* was highly effective when evaluating retinal layer thickness and microanatomical disruptions, such as rosette formation. Our data suggests a more rapid thinning of the ONL and delayed peak of rosette formation in albino *Nrl*^*−/−*^ relative to wildtype albino mice when compared to previous data published in pigmented mice (Roger et al., 2012; Stuck et al., 2012). These differences may partially reflect background strain differences in this comparison, as the albino retina is known to thin at a faster rate than the pigmented counterpart and is less susceptible to age-related cone degeneration (Nadal-Nicolas et al., 2018). Looking beyond the *Nrl*^*−/−*^ model, we believe that there is a natural extension of *InVision* analysis in the context of degenerative models that target any class of retinal cell. For example, optic nerve anatomy is well preserved in *InVision* eyes and immunolabeling for Brn3a in retinal ganglion cells is robust. Collectively, these observations predict that *InVision* imaging and analysis can be used in models of chronic glaucoma, hypertension, or low-vision diseases.

We applied *InVision* to improve our ability to rapidly locate, characterize, and quantify GFP^+^-donor and -acceptor cells after photoreceptor transplantation and to perform an in-depth 3D spatial distribution analysis of donor and acceptor cells to gain insight into the mechanism(s) of MT. Although we performed this analysis in albino recipient mice, our data demonstrate that *InVision* could be applied to pigmented recipient mice and endogenous donor and acceptor reporters may be detected by immunostaining (Figure 3). Our data reveals that donor and acceptor populations exhibit a similar, non-random distribution pattern suggesting that MT is mediated by short-range interactions. Moreover, using a recipient mouse mutant where MT is enhanced, donor cells exhibit a broader distribution within the SRS and a closer proximity to the OLM. These two factors may enhance the short-range interactions made between donor and acceptors, which could explain the enhancement of MT in *Nrl*^*−/−*^ recipients. Finally, consistent with this hypothesis, we show that MT is also enhanced in another mouse recipient with shortened outer segments, the *Prph2*^*−/−*^ mouse (Farjo et al., 2007; Sanyal and Jansen, 1981). Enhancement of MT in the *Nrl*^*−/−*^ recipient is also unlikely due to the cone-like photoreceptors in this genetic model, given that *Prph2*^*−/−*^ retinas have rods and cones. In addition, we cannot exclude the possibility that other abnormalities in the *Nrl*^*−/−*^ and *Prph2*^*−/−*^ retinas contribute to enhanced MT, such as disruption to the OLM and photoreceptor degeneration (Farjo et al., 2007; Schalken et al., 1990; Stuck et al., 2012; Swain et al., 2001). However, we think that latter is unlikely given the difference in the kinetics of degeneration in these models (Schalken et al., 1990; Stuck et al., 2012). In conclusion, the *InVision* protocol facilitates rapid tissue preparation and high-resolution, whole-eye imaging in tumorigenesis, angiogenesis, neurodegeneration, and transplantation models.

### Limitations of the study

The *InVision* approach does, however, have limitations that may be unavoidable within the context of ocular tissue clearing. Due to the crystalline nature of the lens, tissue expansion by CUBIC results in severe lens deformities that produce optical artifacts and inconsistencies (Figure 2B). Enzymatic treatment was used to improve antibody penetration into the retina; however, doing so results in significant digestion of the cornea and sclera that may subsequently hinder analysis of these tissues. Notably, enzymatic digestion may be omitted from this protocol when studying these more superficial structures. Moreover, some antibodies may require further optimization for weakly expressed markers, deeper penetration, or cell-specific labeling. The impact of *InVision*-based processing on the detailed integrity of the retinal pigmented epithelium has not been assessed, making it unclear whether it is suitable for analysis of this structure. Finally, given the limitations in magnification and resolution of LSFM imaging, antigens, or markers with low abundance may not be easily detectable or require additional optimization.

## STAR★Methods

### Key resources table

**Table:** 

REAGENT or RESOURCE	SOURCE	IDENTIFIER
**Antibodies**		

Goat polyclonal anti-GFP	Rockland	Cat#600-101-215; RRID: AB_218182
Goat polyclonal anti-Brn3a	Santa Cruz Biotechnology	Cat#sc-31984; RRID: AB_2167511
Rabbit polyclonal anti-CD31	Abcam	Cat#ab28364; RRID: AB_726362
Goat polyclonal anti-Mrc1	R and D Systems	Cat#Af2535; RRID: AB_2063012
Rabbit polyclonal anti-Laminin	Abcam	Cat#ab11575; RRID: AB_298179
Rabbit polyclonal anti-Cone Arrestin	Millipore	Cat#AB15282; RRID: AB_1163387

**Critical commercial assays**		

Click-iT™ EdU Cell Proliferation Kit	ThermoFisher	Cat#C10338

**Experimental models: Organisms/strains**		

Mouse: *C57BL/6J*	The Jackson Laboratory	Cat# JAX:000664; RRID: IMSR_JAX:000664
Mouse: *Ccdc-136*^*GFP/GFP*^	Smiley et al. (2016)	N/A
Mouse: *Rb*^*loxp*^^*/*^^*loxp*^*; p107*^*-/-*^*;α-Cre*	Chen et al. (2004)	N/A
Mouse: *Rb*^*loxp*^^*/*^^*loxp*^*; p107*^*-/-*^*; p27*^*+/-*^*; α-Cre*	Chen et al. (2004)	N/A
Mouse: *Ndp*^*-/Y*^	MGI	RRID: MGI:4414648
Mouse: *Nrl-GFP*	Mears et al. (2001)	N/A
Mouse: *ROSA*^*mT/mG*^	The Jackson Laboratory	RRID: IMSR_JAX:007576
Mouse: *Nrl*^*-/-*^*;ROSA*^*mT/mG*^	This paper	N/A

**Oligonucleotides**		

See Table 1 for forward and reverse genotyping primer sequences.	N/A	N/A

**Software and algorithms**		

Imaris 9	Bitplane	https://imaris.oxinst.com/
RipleyGUI	Hansson et al. (2013)	N/A
R spatstat	Baddeley and Turner (2005)	N/A
Prism 7	Graphpad	https://www.graphpad.com/scientific-software/prism/

**Other**		

3D printed component CAD files	This paper	N/A

### Resource availability

#### Lead contact

Further information and requests for resources and reagents should be directed to and will be fulfilled by the Lead Contact, Valerie A. Wallace (valerie.wallace@uhnresearch.ca).

#### Materials availability

This study did not generate new unique reagents. There are restrictions to the availability of specific transgenic mouse lines due to the lack of an external centralized repository for its distribution and our need to maintain the stock. We are glad to share these transgenic mice with reasonable compensation by requestor for its processing and shipping.

#### Data and code availability

All datasets are included in the present article and supplementary information. Any additional information required to reanalyze the data reported in this work paper is available from the Lead Contact upon request.

### Experimental model and subject details

#### Mice

All experiments were approved by the University Health Network Research Ethics Board and adhered to the guidelines of the Canadian Council on Animal Care. Animal husbandry was in accordance with the Association for Research in Vision and Ophthalmology (ARVO) Statement for the Use of Animals in Ophthalmic and Vision Research. Animals were maintained under standard laboratory conditions and all procedures were performed in conformity with the University Health Network Animal Care Committee (protocol 3499.16.2). A detailed list of all the mice backgrounds used for each experiment is provided in Table 2. Both male and female wild type and transgenic mice were used in this study except in the case of *Ndp*^*-/Y*^ mice for which only male mice were used. Mouse genotyping was performed by extracting genomic DNA from ear clip samples through incubation in 200 μl alkaline lysis buffer (25 mM NaOH, 0.2 mM EDTA pH 8.0) for 1 h at 95°C. Samples were neutralized with 200 μl neutralization buffer (40 mM Tris-HCl) and genotyped by PCR using primer sets indicated in Table 3.

### Method details

#### InVision permeabilization, depigmentation and clearing

Mice were euthanized by CO_2_ asphyxiation followed by cervical dislocation. Eyes were enucleated with No.5 forceps (Fine Science Tools, Heidelberg, Germany) and placed in a dish with HBSS (Gibco, #14175-095). Extraocular muscles were dissected, and eyes were placed in 2.5% Dispase® II (Sigma, #D4693-1G) solution in HBSS for 30-45 min at 37°C (Figure 1). When spatial orientation of the eye was relevant, the nasal caruncle was not dissected and was used as an anatomical point of reference for the eye. Eyes were then washed in PBS and drop fixed in 4% paraformaldehyde (PFA) overnight at 4°C. For pigmented eyes, samples were immersed in freshly prepared 2.5% H_2_O_2_ (Fischer Scientific, #H325-500) diluted in PBS (Gibco, #10010-023) for 4-8 h at 55°C. Eyes were then immunostained and incubated in CUBIC clearing solutions, as per the original CUBIC protocol (Nojima et al., 2017; Susaki et al., 2014, 2015) with some modifications. Specifically, incubation times were optimized for the size of the rodent eye. Eyes were incubated in 50% CUBIC-1/H_2_O for 2 h at 37°C before an overnight incubation of CUBIC-1 at 37°C followed by a 2-h wash in PBS at RT. Eyes were then incubated in 50% CUBIC-2/PBS for 2 h at 37°C before an overnight incubation of CUBIC-2 at 37°C. Excess CUBIC-2 was removed using a Kimwipe and samples were immersed briefly in Type FF immersion oil (Cargille, #16212) for 10 min at RT.

#### Immunostaining of InVision-treated eyes

After dispase treatment, fixation and depigmentation, eyes were permeabilized and blocked for 1-2 h in 2% TritonX-100 in PBS (PBST) and 2% donkey serum at RT (Figure 1). Eyes were then incubated in primary antibody (Table 1) diluted in 2% PBST and 2% donkey serum for 2 days at RT on a shaker. Samples were then washed in 0.5% PBST 3 times for an hour each at RT on a shaker followed by a secondary antibody incubation in 2% PBST overnight at RT on a shaker. Nucleic acid dye staining (Table 1) was performed simultaneously with the secondary antibody (Table 1). Samples were washed again in PBS 3 times for an hour each before clearing. The full list of antibodies and their respective dilutions can be found in Table 1. Mice were labeled with EdU 24 h prior to sacrifice. Click-iT (ThermoFisher, #C10338) EdU labelling was performed as per the staining kit protocol however eyes were immersed in reaction cocktail overnight at room temperature on a shaker after immunostaining.

#### Immunostaining and clearing of mouse brains

Brains were harvested and immersed in 2.5% Dispase® II solution for 45 min at 37°C following by overnight fixation in 4% PFA overnight. Brain samples not treated with dispase were immediately fixed after harvest. Brains were then washed in PBS and permeabilized with 2% PBST at 37°C overnight. Brains were incubated at 37°C with primary and secondary antibodies in 2% PBST and 5% donkey serum for 7 and 6 days, respectively, with a day of 2% PBST wash in between. Brains were then washed in 2% PBST for a day before dehydration in methanol and clearing as per the iDISCO protocol (Renier et al., 2014).

#### Immunostaining of dissected retinas

1-month-old Ccdc-GFP eyes were harvested, washed in PBS and drop fixed overnight in 4% PFA at 4°C. Intact eyes were either depigmented or depigmentation was not performed. Retinas were then dissected from eyes in PBS and 4 radial cuts were made. For immunostaining, whole-mount retinas were permeabilized and blocked by incubation with 2% PBST and 2% donkey serum for 1 h at RT and then incubated with primary antibodies (Table 1) diluted in 2% PBST and 2% donkey serum for 2 days at RT on a shaker. Retinas were washed three times in 0.5% PBST for 1 h each and incubated overnight at RT with secondary antibodies diluted in 2% PBST. Nuclei were counterstained with SYTOX orange. The retinas were flat-mounted in DAKO mounting media (Cedarlane, #S3023), vitreal side down.

#### Hematoxylin and eosin staining of cleared eyes

For hematoxylin and eosin (H&E) staining, 1-month-old, dispase treated, depigmented and cleared *C57BL/6J* eyes were washed in PBS three times for 1 h each to wash CUBIC-2. Eyes were then incubated in 50% sucrose/PBS overnight at 4°C before freezing in OCT at -80°C. Serial 50 μm thick cryosections were obtained and stained with H&E. H&E staining was performed by first washing slides in PBS for 5 min before immersing slides in hematoxylin for 1 min followed by a tap water rinse. Slides were then immersed in 50%, 70%, 80%, 90%, and 100% ethanol for 1 min each before being dipped three times in eosin followed by a tap water rinse. Slides were then immersed in 50%, 70%, 90% and 100% ethanol for 1 min each again before being immersed in xylene for 5 min followed by mounting in Permount (Fisher Scientific, #SP15-100) and imaged on a Zeiss M2 epifluorescence microscope at 2.5x and 20x magnification.

#### Transplantation of postnatal photoreceptors

P3-P5 *Nrl-GFP* mice were euthanized by decapitation following exposure to CO_2_ for 5 min. Retinas were dissected and collected in CO2-independent media (Thermo Fisher Scientific, #18045088). Retinal tissues were dissociated to single cells with papain (Worthington Biochemical, #LK003150) according to the manufacturer’s directions. Cells were then washed in Ca^2+^/Mg^2+^-free PBS (Millipore, #D8537-500ML), counted using a hemocytometer after staining with a 0.4% Trypan blue viability stain (Thermo Fisher Scientific, #15250061). Cells were then passed through a cell strainer (40 μm) (Thermo Fisher Scientific, #CLS431750) before being re-suspended in retinal explant medium (Ortin-Martinez et al., 2017) supplemented with 0.005% DNAse (Millipore, #D5025- 150KU), Earl’s Balanced Salt Solution (EBSS) (Thermo Fisher Scientific, #14155063) and kept on ice prior to transplantation. Details of the transplantation procedure are provided in (Ortin-Martinez et al., 2017). Briefly, dissociated cells were resuspended at a concentration of 200,000 cells/μL. 1 μL of the cell suspension was delivered using a trans-scleral route into the SRS of 6-8-week old *ROSA*^*mT/mG*^ and *Nrl*^*-/-*^;*ROSA*^*mT/mG*^ adult mice on different experimental days. Transplanted eyes were harvested three weeks after transplantation for analysis.

#### Immunostaining of cryosections from transplanted retinas

Transplanted eyes for cryosection-analysis were harvested and immunostained as previously described (Ortin-Martinez et al., 2017). Briefly, retina cryosections were permeabilized with 0.5% PBST then blocked with 2% donkey serum in 0.5% PBST. Slides were incubated with primary anti-GFP (Table 1) in 2% donkey serum in 0.5% PBST at 1:500 overnight at 4°C. After three washes with PBS, sections were incubated with secondary antibody (Table 1) diluted in 0.5% PBST at 1:500 for 2 h at RT. Slides were washed and stained with Hoechst 33342 (62249, Thermo Fisher Scientific, Mississauga, ON, Canada) at 1:15000 for 20 min at RT. Finally, slides were washed and glass coverslips were mounted with DAKO mounting media (S3023, Cedarlane, Burlington, ON, Canada).

#### Light sheet imaging

Samples were fixed to a sample immersion holder that was modified with a custom, 3D printed insert for stabilizing mouse eyes (Figure S1A). Samples were lowered into 120 ml of imaging oil contained within a crystal imaging cuvette (LaVision BioTec) mounted to an Ultramicroscope II light sheet microscope (LaVision BioTec). Either an Olympus MVPLAPO 2x dry lens equipped with a LaVision BioTec solvent dipping cap, or a Leica HCX APO L20x/0.95 IMM20x solvent-compatible objective lens connected to an infinity-corrected zoom body (LaVision BioTec) was lowered into the oil and focused on the illuminated light sheet plane. A numerical aperture of 0.154 was used with a sheet thickness of 5 μm. Light sheets were generated by an NKT EXW-12, Supercontinuum 1.2-watt laser and emitted signal was acquired with an Andor Neo sCMOS camera. Emission filter sets used were: GFP (525/50 nm), RFP (560/40 nm) or long red (620 nm long pass) (Chroma Technology, Bellows Falls, VT, USA).

#### Multiphoton imaging

A custom 3D printed, 2 chamber specimen-holder (Figure S1B) was designed to allow for cleared tissue samples to be imaged using a Leica HCX PL APO L 20x/1.0 W water immersion lens equipped with a motorized correction collar. Briefly, eyes are immersed in imaging oil (Cargille, #16212) and immobilized in a lower, sealed specimen chamber that is capped by a #1, 22 x 22 mm coverslip (VWR, #16004-094) which separates the lower and upper chambers. An upper chamber is added that is filled with distilled water for use with the Leica HCX PL APO L 20x/1.0 W water immersion lens. Whole eyes were imaged on a dual beam Leica TCS SP8 multiphoton microscope (Leica Microsystems, Wetzlar, Germany) equipped with a 1300nm Chameleon Discovery laser (Coherent, Santa Clara CA, USA). For imaging of GFP, the laser was tuned to 960nm. TdTomato was excited using a fixed-beam laser at 1040nm. Power output for GFP and tdTomato channels was maintained at 45mW. Topro-3 iodide was excited at 1300 nm at 300 mW. Emitted light from all fluorophores was captured using an internal HyD SP GaAsP hybrid detector gated to a range of 500-525 nm for GFP, 550-590 for tdTomato and 630-680 nm for Topro-3. Z-stack images were acquired with 2x accumulation, 2x averaging, 0.70 μm z-resolution and a minimum of 960x960 xy resolution.

#### Confocal imaging

Intact cleared eyes were mounted in a custom-designed 3D-printed chamber (Figure S1C). A #1, 22 x 22 mm coverslip (VWR, #16004-094) was glued to the base of the 3D printed chamber using nail polish. Cleared eyes were mounted in the chamber with the optic nerve placed in the optic nerve slits. The chamber was filled with oil until the eye was at least half-submerged. Dissected whole-mount retinas were flat-mounted after staining prior to imaging. Images were acquired using an LSM 780 (Carl Zeiss Inc. Thornwood, NY, USA) confocal microscope. Images were acquired at a 2048 x 2048 pixel resolution at a maximum of 5% laser intensity and a maximum 800 digital gain. A 10x objective was used for imaging cleared eyes while a 20x objective was used for dissected whole-mount retinas at a 1.0 Airy Unit pinhole size.

### Quantification and statistical analysis

#### Image processing and analysis

MP and LSFM image files were rendered in Imaris 9 (Bitplane, Zurich, Switzerland). When appropriate, 3D images were processed using a background subtraction, followed by a normalization algorithm before making adjustments to brightness and contrast in Surpass mode. Surface rendering, volumetric analysis, and cell (object) identification was performed on LSFM images using the Surface and Spots modules, respectively. All data analysis for surface metrics and object counts were performed using the Vantage module. MP images were only used for qualitative assessments.

#### Analysis of tumor, vasculature and retinal degeneration models

For retinoblastoma eyes, EdU^+^ cells were semi-automatically counted using the Imaris spots function and their relative distribution and density within the eye was visualized using their 3D positional and spot-spot shortest distance data.

For analysis of the vasculature, the surface function of Imaris was used to manually segment the inner retina (outer plexiform layer to retinal ganglion cell layer) and outer retinal (SRS to sclera) vessels. An ∼1 mm region surrounding the optic nerve head was acquired at a 20X magnification on the LSFM and used to semi-automatically determine the volume of the segmented choroidal and inner retinal vasculature.

For retinal degeneration analysis, the surface function of Imaris was used to manually segment the outer nuclear layer (ONL) of 16-20 sections surrounding a ∼2mm region around the optic nerve and compared between genotypes and ages. The number of rosettes were manually counted across the entire eye in *Nrl*^*-/-*^ retinas and compared between ages.

#### Transplantation

To determine the number of GFP^+^ donor and acceptor cells in the SRS and recipient ONL, respectively, the ONL and SRS were manually segmented using the surface function. After 3D segmentation, the spots function was used to semi-automatically count the number of GFP^+^ cells in each segmented region. Transfer efficiency was calculated as the number of GFP^+^ cells in the ONL (acceptor)/ the number of GFP^+^ cells in the SRS (donor) x 100%. This spot analysis was also used to obtain the mean GFP intensity for each cell soma. Transfer intensity was determined as the mean GFP intensity of cells/mean GFP intensity of donor cells x 100%. The difference between the observed, K(t) and expected, E(K(t)), Ripley-K distribution functions of donor and acceptor cells were compared to a distribution following complete spatial randomness (CSR) and to each other using RipleyGUI (Hansson et al., 2013). The G-nearest neighbour distance function for donor and acceptor cells were calculated using the R spatstat package (Baddeley and Turner, 2005). The difference (E(G)-G) between the expected G-function, E(G(r)), and the observed, G(r), were used to compare donor and acceptor populations. To determine the mean distance between donor cells in the SRS and the OLM, a distance transformation was used on the ONL surface in Imaris. The outer edge of the ONL surface object reflected the position of the OLM and the inner most edge reflected the position of the outer plexiform layer in 3D space. Distance transformation of this object provided the shortest distance (μm) of the spot representing a donor cell, to the edge of the ONL surface object (OLM).

#### Quantification of cryosections from transplanted retinas

The number of GFP^+^ cell bodies in the recipient ONL and the number of GFP^+^ cells in the adjacent SRS were counted for every 4^th^ section of a transplanted eye and extrapolated to the entire eye. Transfer efficiency was calculated as the number of GFP^+^ cells in the ONL (acceptor)/ the number of GFP^+^ cells in the SRS (donor) x 100%.

#### Statistical analysis

All statistical analyses were done using GraphPad Prism 7.0 (San Diego, CA, USA). For all tests, differences were considered significant for p < 0.05. For all graphs, ∗ denote p < 0.05, ∗∗ denotes p < 0.01, ∗∗∗ denotes p < 0.001, ∗∗∗∗ denotes p < 0.0001. All means are reported with standard error of the mean unless otherwise noted. For tumor analysis, unpaired Student’s t-test was used to compare tumor volumes between genotypes (*Rb*^*loxp/loxp*^; *p107*^*-/-*^; *p27*^*+/-*^ and *Rb*^*loxp/loxp*^; *p107*^*-/-*^). For vasculature analysis, a 2x2 ANOVA was used with genotype (*C57BL/6J* and *Ndp*^*-/Y*^) and location (choroid and inner retina) as the factors. For neurodegeneration data, a 3x2 ANOVA was used to compare ONL volume with age (1, 3, and 6 months) and genotype (*ROSA*^*mT/mG*^ and *Nrl*^*-/-*^;*ROSA*^*mT/mG*^) as factors. The number of rosettes in *Nrl*^*-/-*^;*ROSA*^*mT/mG*^ mice were compared using a one-way ANOVA with age (1, 3, and 6 months) as the main factor. For transplantation data, Pearson’s correlation coefficients were used to demonstrate linear correlations between the number of GFP^+^ cells in the ONL and SRS. Unpaired Students t-tests were used to compare transfer efficiency, intensity and mean distance to the ONL. RipleyGUI was used to statistically compare RipleyK functions for donor and acceptor populations for each genotype (*ROSA*^*mT/mG*^ and *Nrl*^*-/-*^;*ROSA*^*mT/mG*^). A 2x2x3 (three way) ANOVA was used to compare nearest neighbor distance G-values between genotypes (*ROSA*^*mT/mG*^ and *Nrl*^*-/-*^;*ROSA*^*mT/mG*^), cell type (donor and acceptor) and distance (10, 15 and 20 μm). An unpaired Student’s t-test was used to compare the distance between donor cells and the OLM between genotypes (*ROSA*^*mT/mG*^ and *Nrl*^*-/-*^;*ROSA*^*mT/mG*^). A one-way ANOVA with genotype (*C57BL/6J*, *Nrl*^*-/-*^ and *Prph2*^*-/-*^) as factors was used to compare transfer efficiency.

## Supplemental information

Document S1. Figures S1–S6

Data S1. 3D CAD files for custom-designed and 3D-printed ocular imaging inserts, related to Figure 1

## Footnotes

Supplemental information can be found online at https://doi.org/10.1016/j.isci.2021.102905.
