# Supplementary material for: InVision: An optimized tissue clearing approach for three-dimensional imaging and analysis of intact rodent eyes
Source: iScience. 2021 Jul 26;24(8):102905. doi: 10.1016/j.isci.2021.102905 (PMC8374524; doi:10.1016/j.isci.2021.102905)
Supplement: Document S1. Figures S1–S6 [file mmc1.pdf]

iScience, Volume *24*

Supplemental information

InVision: An optimized tissue clearing approach for three-dimensional imaging and analysis of intact rodent eyes

Akshay Gurdita, Philip E.B. Nickerson, Neno T. Pokrajac, Arturo Ortín-Martínez, En Leh Samuel Tsai, Lacrimioara Comanita, Nicole E. Yan, Parnian Dolati, Nobuhiko Tachibana, Zhongda C. Liu, Joel D. Pearson, Danian Chen, Rod Bremner, and Valerie A. Wallace

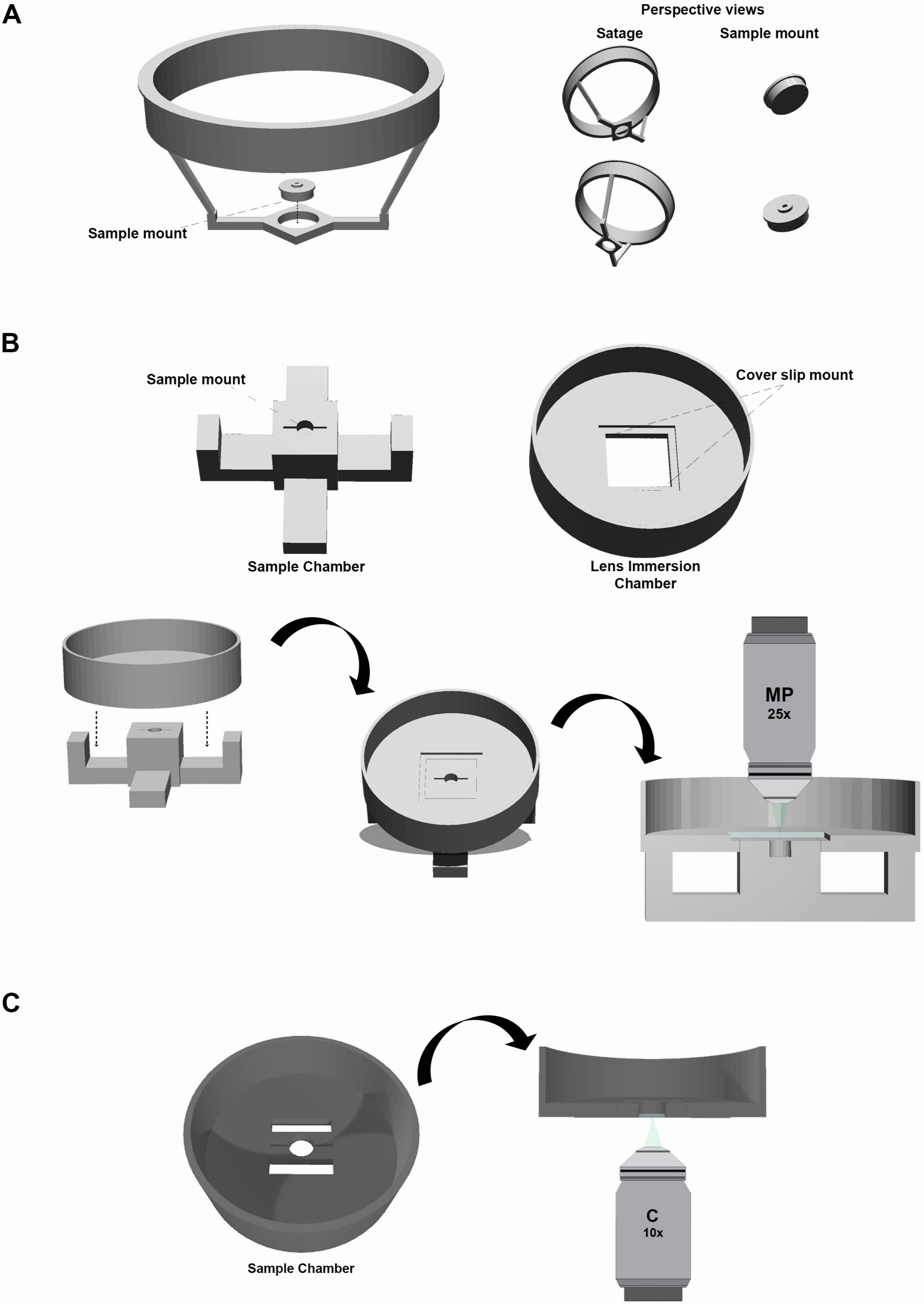
A
Perspective views
Satage
Sample mount
Sample mount
B
Sample mount
Sample Chamber
Cover slip mount
Lens Immersion Chamber
MP
25x
C
Sample Chamber
C
10x

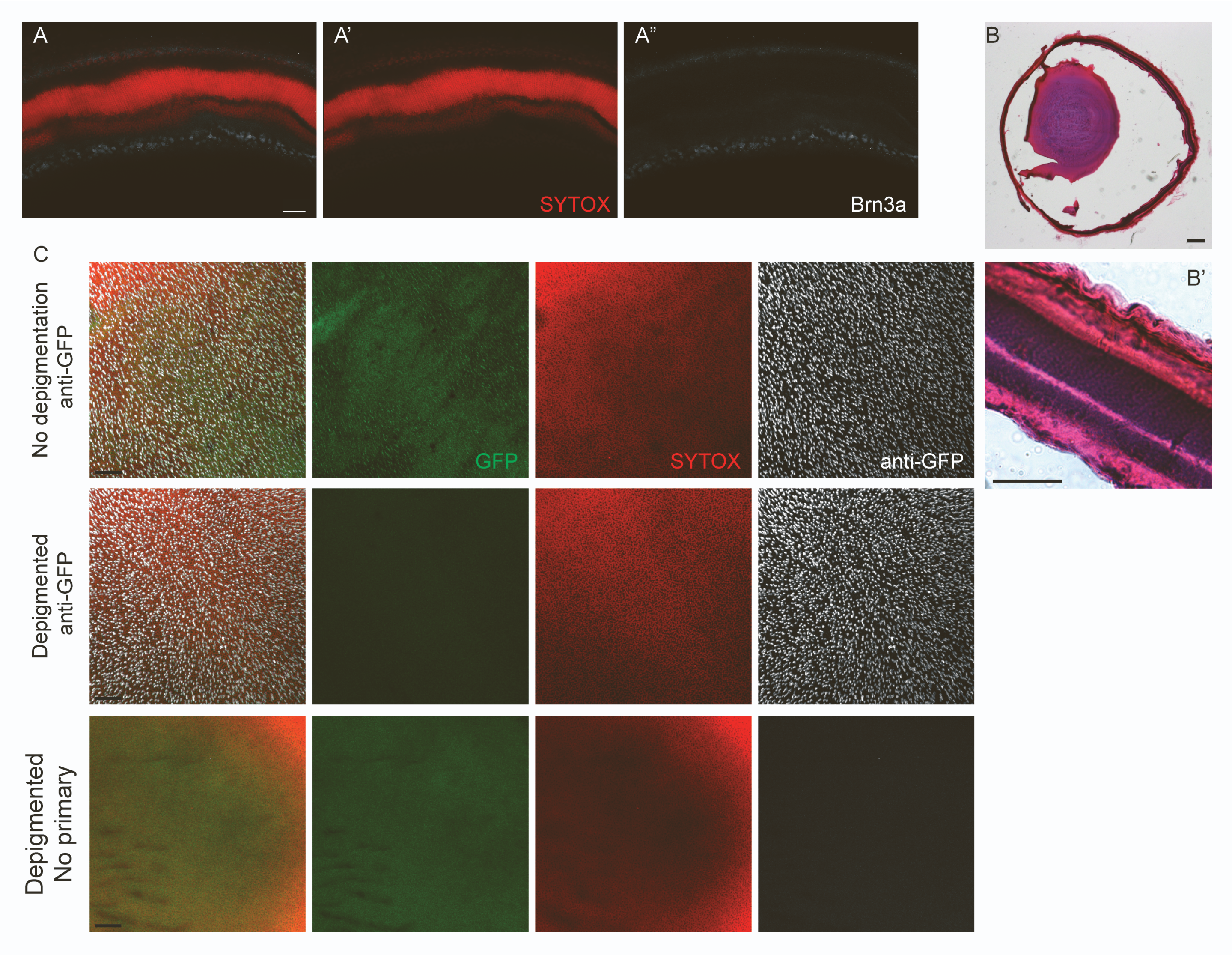

A
A'
A''
SYTOX
Brn3a
B
B'
C
No depigmentation anti-GFP
GFP
SYTOX
anti-GFP
Depigmented anti-GFP
Depigmented No primary

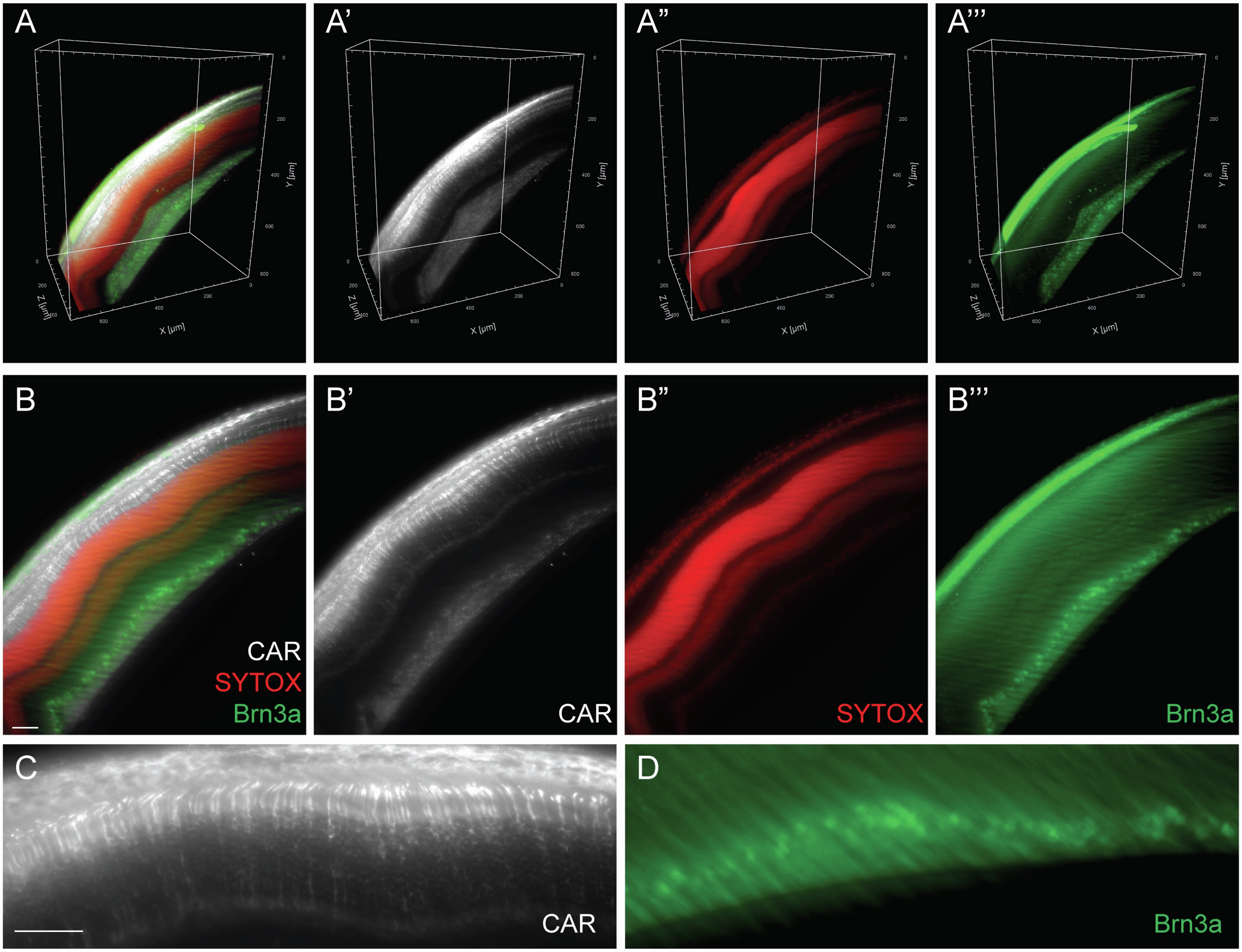
A
A'
A"
A'''
X [µm]
Y [µm]
Z [µm]
B
B'
B"
B'''
CAR
SYTOX
Brn3a
C
D

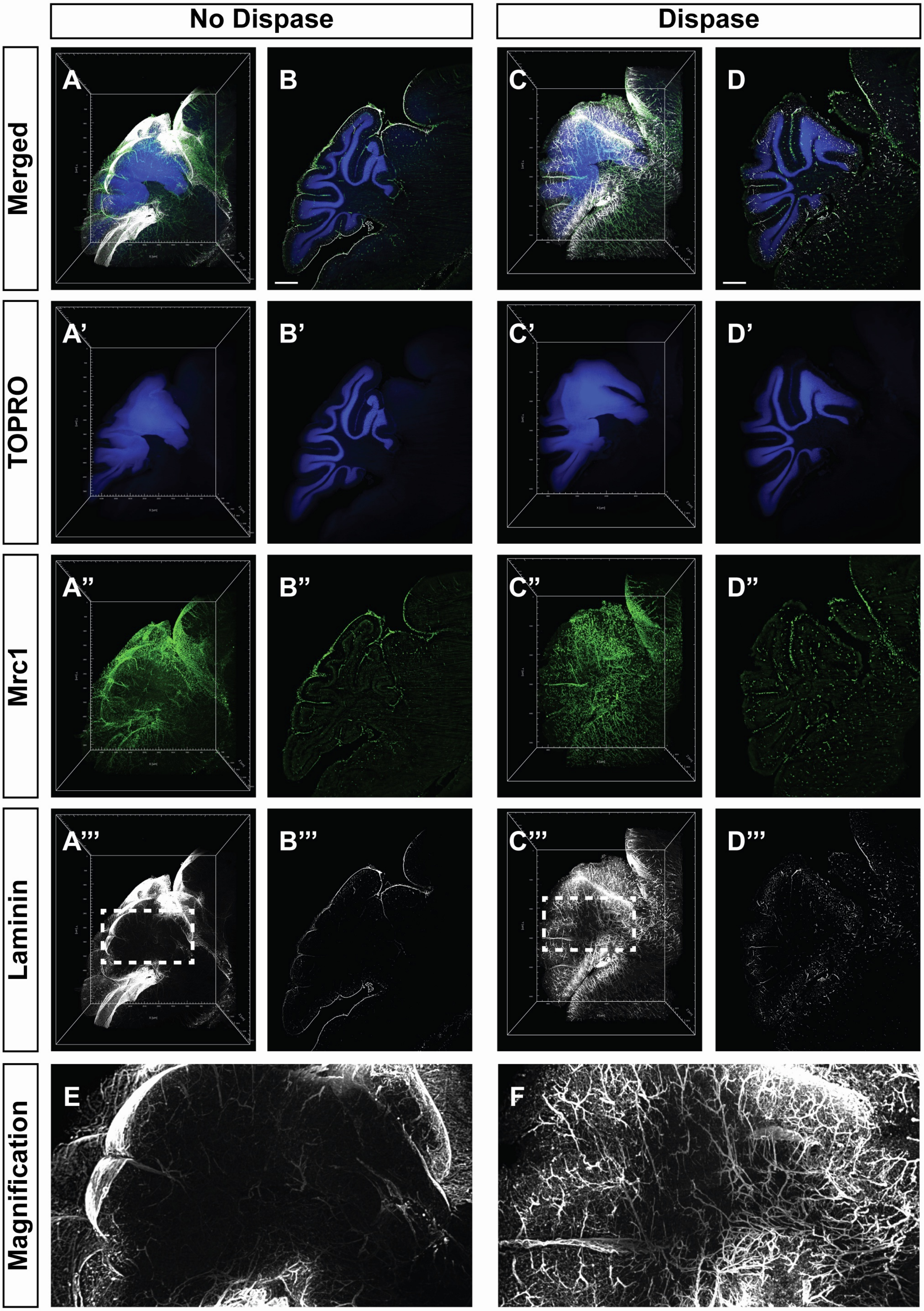
No Dispase
Dispase
Merged
TOPRO
Mrc1
Laminin
Magnification
A
B
C
D
A'
B'
C'
D'
A''
B''
C''
D''
A'''
B'''
C'''
D'''
E
F

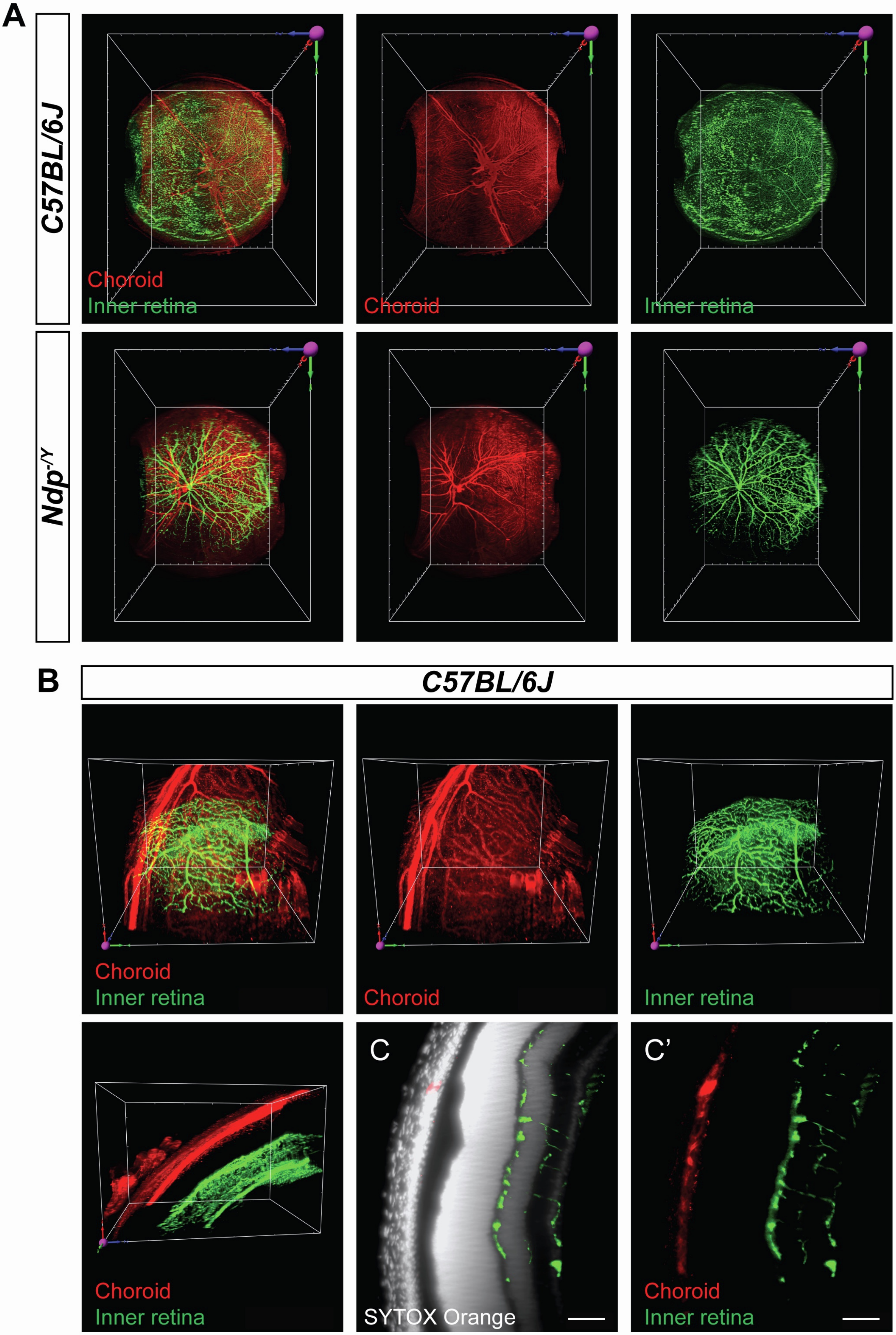

A
C57BL/6J
Choroid
Inner retina
Choroid
Inner retina
$Ndp^{-/Y}$
B
C57BL/6J
Choroid
Inner retina
Choroid
Inner retina
Choroid
Inner retina
C
SYTOX Orange
C'
Choroid
Inner retina

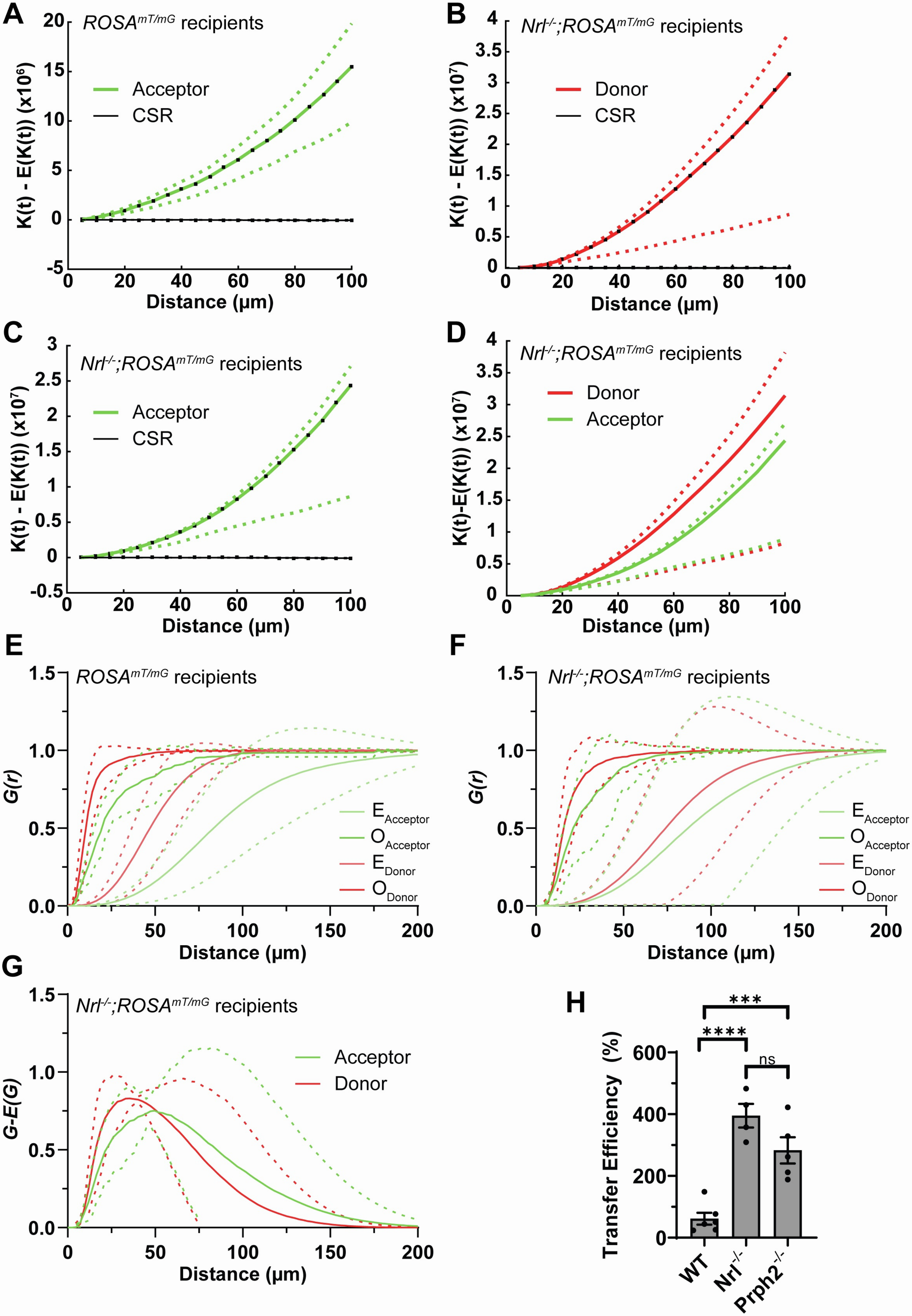

A
ROSA$^{mT/mG}$ recipients
Acceptor
CSR
K(t) - E(K(t)) (x10^6)
Distance (µm)
B
Nrl$^{-/-}$;ROSA$^{mT/mG}$ recipients
Donor
CSR
K(t) - E(K(t)) (x10^7)
Distance (µm)
C
Nrl$^{-/-}$;ROSA$^{mT/mG}$ recipients
Acceptor
CSR
K(t) - E(K(t)) (x10^7)
Distance (µm)
D
Nrl$^{-/-}$;ROSA$^{mT/mG}$ recipients
Donor
Acceptor
K(t)-E(K(t)) (x10^7)
Distance (µm)
E
ROSA$^{mT/mG}$ recipients
G(r)
$E_{Acceptor}$
$O_{Acceptor}$
E_{Donor}
O_{Donor}
Distance (µm)
F
Nrl$^{-/-}$;ROSA$^{mT/mG}$ recipients
G(r)
$E_{Acceptor}$
$O_{Acceptor}$
E_{Donor}
O_{Donor}
Distance (µm)
G
Nrl$^{-/-}$;ROSA$^{mT/mG}$ recipients
G-E(G)
Acceptor
Donor
Distance (µm)
H
Transfer Efficiency (%)
***
****
ns
WT
Nrl$^{-/-}$
Prph2$^{-/-}$

Supplemental Figure Legends

Figure S1. Custom designed 3D printed parts for compatibility with the rodent eye, related to Figure 1.
(A) For the LaVision Ultramicroscope II, an eyeball mount was designed such that the eye is 'cupped' by the mounting stage thus preventing movement while providing the maximal imaging area. **(B)** For MP imaging, eyeballs were mounted in the sample chamber with the optic nerve aligned to the horizontal slots. The sample chamber was also filled with the sample immersion medium (oil) before being capped under the lens immersion chamber. A 22x22 mm coverslip was glued to the coverslip mount on the lens immersion chamber to create a viewing window for the microscope lens and to separate the two chambers. Finally, the lens immersion chamber was filled with the appropriate lens immersion medium, which in this case was water. **(C)** For confocal imaging, a 22x22 mm coverslip was glued to the bottom of the 3D printed chamber to create a viewing window for an inverted microscope objective. Eyes are mounted in the sample chamber with the optic nerves aligned to the horizontal slots and the chamber was filled with the oil immersion media for imaging.

Figure S2. Dispase treatment of intact eyes does not significantly affect ocular structure, related to Figure 2 and 3.
(A-A") Confocal imaging, using a custom-designed 3D chamber, of cleared, intact, dispase-treated and depigmented *C57BL/6J* eyes after anti-Brn3a immunostaining. Images were obtained at a depth of ~400 μm from the bottom of the tissue. **(B)** Low and **(B')** high magnification hematoxylin and eosin stained 50 μm thick cryosections, of dispase-treated, depigmented and cleared *C57BL/6J* eyes. **(C)** Dissected retinas from non-depigmented or depigmented *Ccdc-GFP* eyes. Secondary donkey anti-goat IgG 647 was used against goat anti-GFP (white) primary antibody to also visualize endogenous GFP (green) expression. SYTOX orange was used as a nuclear marker (red). Depigmentation results in endogenous GFP quenching but is recoverable by anti-GFP immunostaining. Unlike intact eyes, dissected retinas can be immunostained without dispase treatment. Scale bars = 50 μm for A, B' and C, 200 μm for B.

Figure S3. InVision is amenable to double-immunostaining of dispase treated, depigmented, *C57BL/6J* eyes, related to Figure 3.
(A) 3D reconstruction and **(B)** 2D cross sections of a dispase treated, depigmented *C57BL/6J* retina double immunostained for anti-cone arrestin (CAR; white), anti-Brn3a (green) and SYTOX orange (red). **(C)** and **(D)** High magnification region of B' and B'''. Scale bar = 50 μm for B, C and D.

Figure S4. Dispase treatment of *C57BL/6* brains improves antibody penetration in cleared cerebellum, related to Figure 3.
(A, C) 3D reconstructions and **(B, D)** 2D cross sections of *C57BL/6J* mouse brains at post-natal day 14 immunostained with TOPRO (blue), Mrc1 (green) and Laminin (white). **(C, D)** Dispase-treated brains demonstrate improved laminin and Mrc1 antibody penetration compared to **(A, B)** non-dispase treated brains. **(E, F)** High magnification regions of the 3D reconstructed **(A''')** non-dispase treated and **(C''')** dispase treated brains. Laminin staining, in non-dispase treated

brains is restricted to the outer surface of the cerebellum compared to broad cerebellar immunogenicity in dispase-treated brains.

Figure S5. Additional viewing perspectives and imaged regions of *C57BL/6J* and *Ndp*$^{-/Y}$ retinal vasculature, related to Figure 5.
(A) 3D reconstruction of choroidal and inner retinal vessels along the XZ plane for *C57BL/6J* and *Ndp*$^{-/Y}$ retinas immunostained with anti-CD31 antibodies. **(B)** 3D reconstruction and **(C-C')** cross section of choroidal and inner retinal peripheral vessels at 20X magnification in *C57BL/6J* retinas stained with anti-CD31. Scale bar = 100 µm.

Figure S6. Ripley K and Nearest neighbour distance G functions for wild type and *Nrl*$^{-/-}$ retinas, related to Figure 9.
(A-C) Comparison of the difference, K(t)-E(K(t)), in observed, K(t), and expected, E(K(t)), Ripley K distribution functions to complete spatial randomness (black line). The mean distribution of **(A)** acceptor cells in wild-type recipients, **(B)** donor cells in *Nrl*$^{-/-}$ recipients and **(C)** acceptor cells in *Nrl*$^{-/-}$ recipient retinas. **(D)** Comparison of the difference in observed and expected Ripley K functions between donor and acceptor cell populations in *Nrl*$^{-/-}$ recipient retinas. Comparison of the observed (O) and expected (E) nearest neighbour distance (G) functions for donor and acceptor populations in **(E)** wild type and **(F)** *Nrl*$^{-/-}$ recipients. **(G)** Comparison of the difference in observed and expected G functions between donor (red) and acceptor (green) populations. Dashed lines in A-G represent the 95% confidence intervals. **(H)** Comparison of the transfer efficiency in wild type, *Nrl*$^{-/-}$ and *Prph2*$^{-/-}$ recipients as determined by quantification of serial sections. Data are represented as means ± SEM. *** $p < 0.001$, **** $p < 0.0001$. One-way ANOVA with Tukey's post-hoc multiple comparisons test with genotype (*C57BL/6J*, *Nrl*$^{-/-}$ and *Prph2*$^{-/-}$) as factors.
